# Toward exact predictions of spin-phonon relaxation times: An ab initio implementation of open quantum systems theory

**DOI:** 10.1126/sciadv.abn7880

**Published:** 2022-08-05

**Authors:** Alessandro Lunghi

**Affiliations:** School of Physics, AMBER and CRANN Institute, Trinity College, Dublin 2, Ireland.

## Abstract

Spin-phonon coupling is the main driver of spin relaxation and decoherence in solid-state semiconductors at finite temperature. Controlling this interaction is a central problem for many disciplines, ranging from magnetic resonance to quantum technologies. Spin relaxation theories have been developed for almost a century but often use a phenomenological description of phonons and their coupling to spin, resulting in a nonpredictive tool and hindering our detailed understanding of spin dynamics. Here, we combine time-local master equations up to the fourth order with advanced electronic structure methods and perform predictions of spin-phonon relaxation time for a series of solid-state coordination compounds based on both transition metals and lanthanide Kramers ions. The agreement between experiments and simulations demonstrates that an accurate, universal, and fully ab initio implementation of spin relaxation theory is possible, thus paving the way to a systematic study of spin-phonon relaxation in solid-state materials.

## Teaser

Ab initio open quantum system theory can predict spin-phonon relaxation time in solid-state magnetic systems.

## INTRODUCTION

Spin is a fundamental property of particles such as electrons and protons, and it is responsible for many emergent properties of materials, including fundamental ones such as the chemical bond and magnetic phenomena. Thanks to its natural weak coupling to other degrees of freedom, electron’s spin also represents a prototypical quantum system that can be coherently driven for long times. The establishment of thermal equilibrium in localized spin moments in the condensed phase, a process called relaxation, is mainly driven by three interactions: spin-spin, spin-conduction electron, and spin-phonon coupling (*1*). In the case of magnetically diluted semiconductors at temperatures above a few kelvins, the latter is the most important one. In a nutshell, the coupling between spin and phonons is possible thanks to relativistic interactions, such as the spin-orbit one, which couples the spin degrees of freedom to the electrostatic potential of the nuclei. Spin can thus be seen as an open system, where the oscillatory thermal motion of atoms acts as a time-dependent perturbation that leads to spin thermalization and decoherence.

On the one hand, a full understanding of spin-phonon relaxation and spin-phonon coupling has the potential to open up previously unidentified ways to address central problems for modern technologies such as magnetic information storage (*2*), spintronics (*3*), quantum information science (*4*, *5*), magnetic resonance (*6*), and more. On the other hand, the study of spin relaxation in the condensed phase represents a fundamental benchmark for our understanding of the theory of open quantum systems. Although a formally exact description of the quantum dynamics of open systems is achievable through time- convolutionless (*7*, *8*) or Nakajima-Zwanzig (*9*) equations, their application is often done either in a parametric fashion or for simplified models, therefore escaping the ultimate benchmark of a full ab initio implementation for realistic systems. Spin-phonon coupling represents a paradigmatic example of open quantum system dynamics and offers a unique test bed for open quantum system theory and ab initio simulations at the same time.

Spin-phonon relaxation has now been debated for almost a century starting with the seminal works of Waller (*10*), Van Vleck (*11*), Redfield (*12*), and Orbach (*13*). Redfield’s relaxation theory and its adaptations form the fundamental building block of our understanding of spin relaxation and have served as the basis to model both nuclear and electronic spin relaxation experiments. Redfield’s theory is generally adapted to the case of solid-state systems by accounting for a Debye-like phonon density of state and by including all the details about spin-phonon coupling under a single average phenomenological coupling constant (*13*). Despite the incredible usefulness of such a formalism for interpreting experiments, these approximations have the major drawback of making it impossible to use the theory as a real predictive tool. Only recently, fully ab initio methods to predict spin-phonon relaxation in solid-state materials have been proposed (*14*–*19*). At the heart of these methods, there is a combination of Redfield’s theory of relaxation and electronic structure methods. The latter are used to define all the coefficients needed to set up the former on a material-specific case and thus enable a nonparametric treatment of all the relevant interactions, such as spin and phonon spectra, and all the spin-phonon coupling coefficients. Applications of this method to magnetic molecules have recently generated large interest, with examples of studies in both molecular qubits (*14*, *17*, *20*–*23*) and single-ion magnets (*15*, *16*, *24*–*30*). Coordination compounds offer a versatile playground to test relaxation theories as their chemical structure and properties can be finely controlled and characterized experimentally (*31*, *32*). At the same time, they allow for the use of advanced electronic structure methods to accurately describe their lattice and magnetic properties (*33*–*35*).

Despite recent achievements in this field, a fully quantitative prediction of relaxation times has remained elusive until now, and deviations of orders of magnitude with respect to experimental data are commonly observed. Such a situation casts shadows on our understanding of spin-phonon relaxation and on the reliability of ab initio methods for blind predictions of spin relaxation time in the absence of experimental validation.

**Fig. 1.**
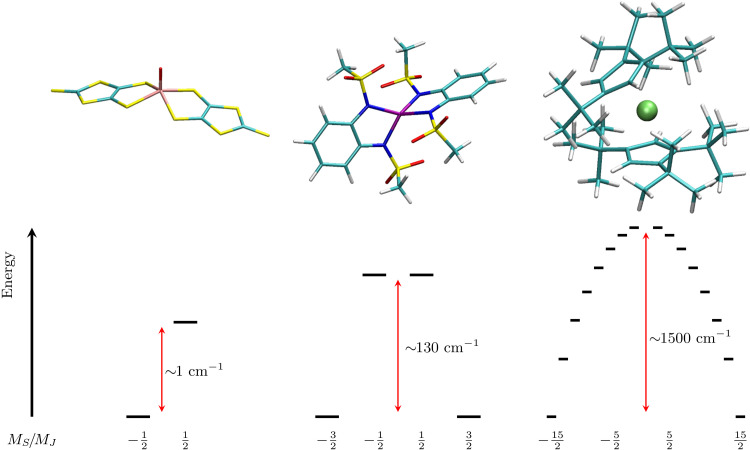
Molecular structures. (**Top**) Left, middle, and right panels show the structures of **(1)**, **(2)**, and **(3)**, respectively. Color code: Pink for vanadium, purple for cobalt, light green for dysprosium, green for carbon, blue for nitrogen, red for oxygen, yellow for sulfur, and white for hydrogen. (**Bottom**) Left, middle, and right panels show the spin states’ energy splitting for **(1)** in a field of **∼**1 T and for **(2)** and **(3)** in the absence of external field, respectively.

In this contribution, we advance the field of ab initio spin dynamics by demonstrating that quantitative predictions of spin relaxation rate can be obtained for realistic solid-state compounds. We review the derivation of the dynamical equations for the spin-reduced density matrix in solid-state materials (*17*, *22*, *27*) and individuate the key importance of (i) comparing simulations with measurements done on magnetically diluted samples, (ii) including two-phonon contributions stemming from either the second- or the fourth-order perturbation theory, and (iii) accounting for the dynamics of the entire density matrix, i.e., including the explicit description of coherence terms’ dynamics. We perform simulations for three systems: **(1)** an *S* = 1/2 molecular qubit [VO(dmit)_2_]^2−^ [dmit = (1,3-dithiole-2-thione-4,5-dithiolate)] (*36*), **(2)** an *S* = 3/2 mononuclear complex [CoL_2_]^2−^ {H_2_L = [1,2-bis(methanesulfonamido)benzene]} (*37*, *38*), and **(3)** a *J* = 15/2 mononuclear complex ${\left[{\text{DyCp}}_{2}^{\text{ttt}}\right]}^{+}\;{\text{Cp}}^{\text{ttt}}=([C_{5}{H_{5}}^{t}{\text{Bu}}_{3}-1,2,4])$ (*16*). The molecular structures of **(1)** to **(3)** are reported in Fig. 1. These compounds represent the state of the art in molecular magnetism for what concerns molecular qubits and single-molecule magnets with long spin lifetime. Moreover, their properties span a large range of magnetic splitting, as depicted in Fig. 1, and relaxation times (vide infra), thus providing an extremely robust proof of concept that ab initio spin dynamics can now be used to predict and understand spin relaxation in solid-state magnetic systems.

## RESULTS

### Ab initio theory of spin-phonon relaxation

A quantum mechanical description of spin-phonon relaxation requires the description of all the players involved: the spin, the phonons, and their interaction. The total Hamiltonian operator is thus

$$\hat{H}={\hat{H}}_{s}+{\hat{H}}_{\text{ph}}+{\hat{H}}_{s-\text{ph}}$$ (1)

where the first two terms correspond to the spin and phonon Hamiltonians, respectively, and the third one represents the coupling between the two subsystems. The spin Hamiltonian is chosen to describe the low-lying electronic states of the real all-electron system according to well-defined and accurate mapping strategies (*33*, *34*). The phonon Hamiltonian is chosen as a simple sum of harmonic oscillators. Under a weak-coupling assumption, the interaction between a spin system and an ensemble of phonons, *q*_α_, can be modeled with a spin-phonon coupling Hamiltonian of the form

$${\hat{H}}_{s-\text{ph}}=\sum_{\alpha }{\left(\frac{\partial {\hat{H}}_{s}}{\partial q_{\alpha }}\right)}_{0}q_{\alpha }(t)+\sum_{\alpha \geq \beta }{\left(\frac{\partial^{2}{\hat{H}}_{s}}{\partial q_{\alpha }\partial q_{\beta }}\right)}_{0}q_{\alpha }(t)q_{\beta }(t)$$ (2)

where the summations are understood to run over both phonon band indexes and reciprocal lattice *q*-points.

The spin-reduced density matrix in the interaction picture (*7*, *8*), ${\hat{\rho }}_{s}$, describes the state of the spin under the effect of the interaction with the phonon bath, and the formalism of time-convolutionless master equations provides an exact description of its time evolution (*7*, *8*)

$$\frac{d{\hat{\rho }}_{s}}{\mathrm{dt}}=\hat{\hat{R}}(t){\hat{\rho }}_{s}(t)$$ (3)

The super-operator $\hat{\hat{R}}(t)$ describes how the density operator evolves in time, and it contains all the information about the phonons’ dynamics and their effect on the spin. If all the terms of Eq. 1 are known, then Eq. 3 can then, in principles, be solved exactly. However, to numerically solve Eq. 3, $\hat{\hat{R}}(t)$ is often systematically expanded in a perturbative way (*7*, *8*), $\hat{\hat{R}}(t)=(\hat{\hat{R}}2(t)+\hat{\hat{R}}4(t)+\ldots )$, where contributions become progressively less important. Further simplifications are also commonly performed. For instance, assuming a separation of time scales between spin relaxation and the intrinsic relaxation rates of the phonons, it is possible to assume that the latter are always at thermal equilibrium and therefore apply the Born-Markov approximation. Namely, we assume that phonons have a much larger specific heat than the spin system [no phonon bottleneck (*39*)] and that they are able to rapidly equilibrate through anharmonic phonon-phonon scattering while at the same time exchanging energy with the spin.

Under these assumptions and considering only the second-order contribution $\hat{\hat{R}}2(t)$, Eq. 3 becomes identical to the well-known Redfield equations (*17*)

$$\frac{d\rho_{\mathrm{ab}}^{s}(t)}{\mathrm{dt}}=\sum_{n}\;\sum_{\mathrm{cd}}e^{i(\omega_{\mathrm{ac}}+\omega_{\mathrm{db}})t}R2_{\mathrm{ab},\mathrm{cd}}^{n-\text{ph}}\rho_{\mathrm{cd}}^{s}(t)$$ (4)

which describe how each element of the density matrix changes in time and the superscript *n*–ph describes the number of phonons simultaneously involved during this process. Diagonal elements of the density matrix, $\rho_{\mathrm{aa}}^{s}$, describe the population of each spin state, while out-of-diagonal terms, $\rho_{\mathrm{ab}}^{s}$ with *a* ≠ *b*, describe the coherence of the system, the hallmark of quantumness.

**Fig. 2.**
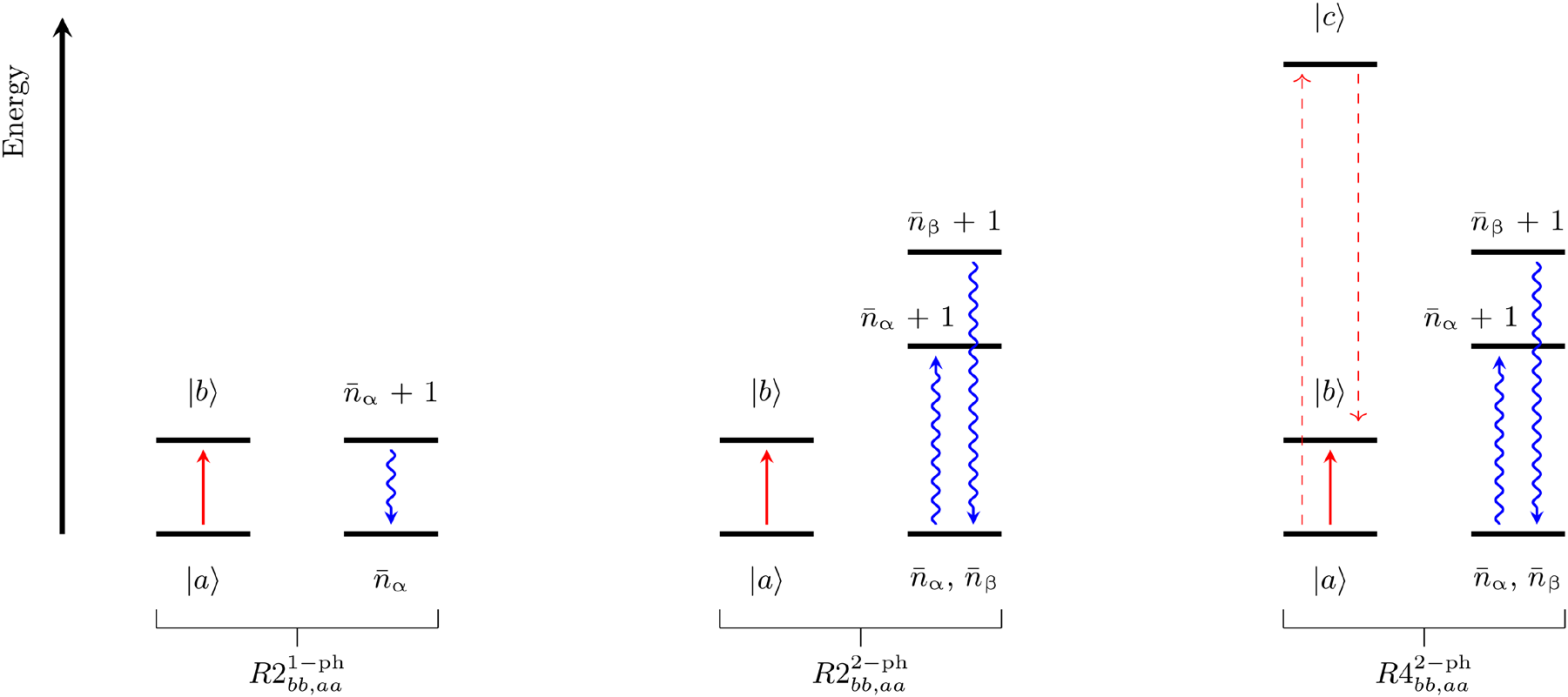
Schematic representation of spin-phonon transitions. (**Left**) A spin transition, from the state ∣*a*〉 to the state ∣*b*〉, due to a resonant one-phonon process as dictated by the super-operator *R*2^1 − ph^. (**Middle**) A spin transition among the same states but mediated by a two-phonon process, where one phonon is emitted and one is simultaneously absorbed. This is a process accounted for by the super-operator *R*2^2 − ph^. (**Right**) A spin transition among the same states induced by the super-operator *R*4^2 − ph^, where a two-phonon process can occur thanks to virtual transitions (red dashed lines) to an excited spin state ∣*c*〉.

Considering only the linear term of Eq. 2, $\hat{\hat{R}}2^{n-\text{ph}}$ becomes (*a* ≠ *c* and *b* ≠ *d*)

$$R2_{\mathrm{ab},\mathrm{cd}}^{1-\text{ph}}=\frac{\pi }{2\hbar^{2}}\sum_{\alpha }\{V_{\mathrm{ac}}^{\alpha }V_{\mathrm{db}}^{\alpha }G^{1-\text{ph}}(\omega_{\mathrm{bd}},\omega_{\alpha })+V_{\mathrm{ac}}^{\alpha }V_{\mathrm{db}}^{\alpha }G^{1-\text{ph}}(\omega_{\mathrm{ac}},\omega_{\alpha })\}$$ (5)

where the terms $V_{\mathrm{ab}}^{\alpha }$ are a short-hand notation for $\langle a|(\partial {\hat{H}}_{s}/\partial q_{\alpha })|b\rangle$, and ∣*a*> and ∣*b*> are eigenstates of ${\hat{H}}_{s}$. Last, ω*_ab_* = (*E_a_* − *E_b_*)/ħ, where *E_a_* and *E_b_* are eigenvalues of ${\hat{H}}_{s}$. The full expression of Eq. 5 is reported in the Supplementary Materials. The function *G*^1 − ph^ that appears in Eq. 5 is the Fourier transform of the single-phonon correlation function and reads

$$G^{1-\text{ph}}(\omega ,\omega_{\alpha })=\delta (\omega -\omega_{\alpha }){\bar{n}}_{\alpha }+\delta (\omega +\omega_{\alpha })({\bar{n}}_{\alpha }+1)$$ (6)

where ${\bar{n}}_{\alpha }={\left[\text{exp}(\hbar \omega_{\alpha }/k_{B}T)-1\right]}^{-1}$ is the Bose-Einstein distribution describing phonons’ thermal population, ħω_α_ is the α-phonon energy, and *k*_B_ is the Boltzmann constant. The function *G*^1 − ph^ accounts for the spectral density and population of phonons. Equations 4 and 5 describe one-phonon resonant spin transitions (see Fig. 2). The one-phonon transitions described by Eq. 5 can account for either the direct or the Orbach relaxation mechanism, where the former involves a single transition between spin states with opposite polarization (*M*_s_ = ±*S*) and the latter instead involves transitions to states with intermediate spin polarization.

Considering now the quadratic term of Eq. 2, $\hat{\hat{R}}2^{n-\text{ph}}$ becomes (*22*) (*a* ≠ *c* and *b* ≠ *d*)

$$\begin{array}{c} R2_{\mathrm{ab},\mathrm{cd}}^{2-\text{ph}}=\frac{\pi }{4\hbar^{2}}\sum_{\alpha \geq \beta }\{V_{\mathrm{ac}}^{\alpha \beta }V_{\mathrm{db}}^{\alpha \beta }G^{2-\text{ph}}(\omega_{\mathrm{bd}},\omega_{\alpha },\omega_{\beta })+\\ V_{\mathrm{ac}}^{\alpha \beta }V_{\mathrm{db}}^{\alpha \beta }G^{2-\text{ph}}(\omega_{\mathrm{ac}},\omega_{\alpha },\omega_{\beta })\} \end{array}$$ (7)

where $V_{\mathrm{ab}}^{\alpha \beta }$ now stands for $\langle a|(\partial^{2}{\hat{H}}_{s}/\partial q_{\alpha }\partial q_{\beta })|b\rangle$. The full expression of Eq. 5 is reported in the Supplementary Materials. The function *G*^2 − ph^ accounts for three possible processes involving two phonons: absorption of two phonons, emission of two phonons, and simultaneous emission of one phonon and absorption of a second one. Here, we only report the latter contribution, which reads

$$G^{2-\text{ph}}(\omega_{\mathrm{ba}},\omega_{\alpha },\omega_{\beta })=\delta (\omega_{\mathrm{ba}}-\omega_{\alpha }+\omega_{\beta }){\bar{n}}_{\alpha }({\bar{n}}_{\beta }+1)$$ (8)

As evident from Eq. 8, in two-phonon transitions, only the sum or difference of the two phonons’ energies must be resonant with the spin transition, while there is no constraint on the energy of the single vibrations. Spin relaxation mechanisms due to two-phonon processes generally fall under the name of Raman relaxation mechanism, and those arising from Eq. 7 are schematically represented in the middle panel of Fig. 2.

At this point of the derivation of the Redfield equations, the secular approximation is generally performed. This approximation involves setting to zero the matrix elements of $R2_{\mathrm{ab},\mathrm{cd}}^{2-\text{ph}}$ for which (ω*_ac_* + ω*_db_*) ≠ 0. This is justified by the fact that the terms (ω*_ac_* + ω*_db_*) appear as arguments of an oscillatory term in Eq. 4. These oscillating terms are averaged out for times commensurable to the spin relaxation time, τ, provided that the period of the natural spin oscillations (ω*_ab_*) is much shorter than τ. Bearing in mind that spin-phonon relaxation in magnetic molecules often occurs on time scales of nanoseconds to hundreds of seconds, depending on the value of *T* and the specific system, the secular approximation is generally fulfilled for systems with zero-field splitting or in the presence of small external or dipolar magnetic fields. Under these conditions, only the terms $R2_{\mathrm{aa},\mathrm{bb}}^{n-\text{ph}}$ and $R2_{\mathrm{ab},\mathrm{ab}}^{n-\text{ph}}$, namely, population transfer and coherence relaxation terms, contribute to the dynamics of ${\hat{\rho }}_{s}$. However, when dealing with Kramers systems in zero external magnetic field, additional terms of $R2_{\mathrm{ab},\mathrm{cd}}^{2-\text{ph}}$ also survive the secular approximation in virtue of the spectrum degeneracy that makes additional terms (ω*_ac_* + ω*_db_*) vanish. For instance, the population-coherence transfer process $R2_{\mathrm{aa},\mathrm{bc}}^{n-\text{ph}}$, with *E_b_* = *E_c_*, and coherence transfer terms $R2_{\mathrm{ad},\mathrm{bc}}^{n-\text{ph}}$, with *E_b_* = *E_c_* and *E_a_* = *E_d_*, would not be washed out by the secular approximation and could, in principle, contribute to the spin-phonon dynamics (*40*, *41*).

Extending the expansion of $\hat{\hat{R}}(t)$ up to the fourth order and considering only the linear term of spin-phonon coupling, we obtain another source of two-phonon spin transitions. A set of equations describing the diagonal population terms of ${\hat{\rho }}_{s}$ due to this contribution reads (*8*)

$$\frac{d\rho_{\mathrm{aa}}^{s}(t)}{\mathrm{dt}}=\sum_{b}R4_{\mathrm{aa},\mathrm{bb}}^{2-\text{ph}}\rho_{\mathrm{bb}}^{s}(t)$$ (9)

${\hat{\hat{R}}4}^{2-\text{ph}}$ receives contributions from the same two-phonon processes as ${\hat{\hat{R}}2}^{2-\text{ph}}$. Here, we explicitly discuss the sum/difference one and refer to the Supplementary Materials for the complete expression of ${\hat{\hat{R}}4}^{2-\text{ph}}$ and a discussion on its derivation and implementation subtleties. For this contribution, ${\hat{\hat{R}}4}^{2-\text{ph}}$ reads

$$R4_{\mathrm{aa},\mathrm{bb}}^{2-\text{ph}}=\frac{\pi }{2\hbar^{2}}\sum_{\alpha \geq \beta }{|\sum_{c}\frac{\langle a|{\hat{V}}_{\alpha }|c\rangle \langle c|{\hat{V}}_{\beta }|b\rangle }{E_{c}-E_{b}+\hbar \omega_{\beta }}+\frac{\langle a|{\hat{V}}_{\beta }|c\rangle \langle c|{\hat{V}}_{\alpha }|b\rangle }{E_{c}-E_{b}-\hbar \omega_{\alpha }}|}^{2}G^{2-\text{ph}}(\omega_{\mathrm{ba}},\omega_{\alpha },\omega_{\beta })$$ (10)

Differently from Eq. 7, the factor *G*^2 − ph^ in Eq. 10 is multiplied by a prefactor accounting for the presence of excited spin states of energy *E_c_*, i.e., the virtual states often invoked in perturbation theory. The processes described by Eq. 10 also contribute to the so-called Raman relaxation and are depicted in the right panel of Fig. 2. Equation 10 differs from a previously presented one, where not all the terms had been included (*27*, *30*). Last, we note that Eq. 10 does not account for the dynamics of coherence terms, and it is therefore only strictly valid for nondegenerate spin spectra, where coherence and population terms are decoupled.

As long as the Born-Markov and secular approximations hold, the equations just detailed provide an exact description of spin relaxation up to two-phonon processes. The only remaining challenge to their implementation lies in the definition of the many coefficients that enter these equations. This challenge is tackled with electronic structure methods. Methods such as complete active space self-consistent field (CASSCF) and density functional theory (DFT) have now reached a high degree of sophistication in the prediction of spin Hamiltonian parameters of magnetic molecules (*33*, *34*) as well to predict phonons’ frequencies and normal modes of vibrations (*42*).

### Spin-phonon relaxation in *S* = 1/2 systems

Compound **(1)** is a V^4+^ coordination complex with an *S* = 1/2 ground state and a nuclear spin *I* = 7/2 (*36*). The spin Hamiltonian can be modeled as

$${\hat{H}}_{s}=\mu_{B}\vec{B}\cdot g\cdot \vec{S}+\gamma_{N}\vec{B}\cdot \vec{I}+\vec{S}\cdot A\cdot \vec{I}$$ (11)

where μ_B_ is the electron’s Bohr magneton, **g** is the effective electronic Landè tensor, γ_N_ is the nuclear gyromagnetic factor of ^51^V, and **A** is the hyperfine coupling tensor. Compound **(1)** crystallizes in a monoclinic unit cell containing four molecular units and eight tetraphenylphosphonium counter ions. The entire unit cell was optimized with periodic DFT (pDFT) and used for a Γ-point phonon calculation as detailed in Materials and Methods. The vibrational density of states is reported in fig. S1. The tensors **g** and **A** were computed with DFT after the structural optimization. Predicted values of (*g_xx_*, *g_yy_*, *g_zz_*) = (1.976,1.986,1.988) are in perfect agreement with experimental ones (*36*). Computed values of **A** are found sensitive to the choice of scalar relativistic corrections used in the DFT simulation, with zeroth order regular approximation (ZORA) underestimating the values up to 23% and Douglas-Kroll-Hess (DKH) approximation overestimating them up to 38%. All the terms of the spin-phonon coupling Hamiltonian of **(1)**, including second-order terms, are computed by taking the numerical derivative of all the parameters in Eq. 11 that depend on molecular coordinates and affect spin dynamics, namely, **g** and **A**. Notably, here we perform the calculation of second-order spin-phonon coupling coefficients fully ab initio and without using any machine learning interpolator as done in previous work (*22*). We neglect the contributions of Eq. 5 to relaxation, which are known to lead to direct relaxation (*17*). The latter only overcome two-phonon relaxation for *T* < 10 K (*22*, *36*). In that regime, experimental values of τ are affected by spin diffusion, which would make the comparison with simulations not straightforward. For **(1)**, we also exclude the contributions coming from Eq. 10. This is justified by the fact that a spin 1/2 is a two-level system and lacks excited spin states able to act as virtual states in Eq. 10. In principle, one could include transitions to higher electronic excited states. However, in **(1),** these states lie 20,000 cm^−1^ above the ground state (*20*), far away from any phonon excitation.

**Fig. 3.**
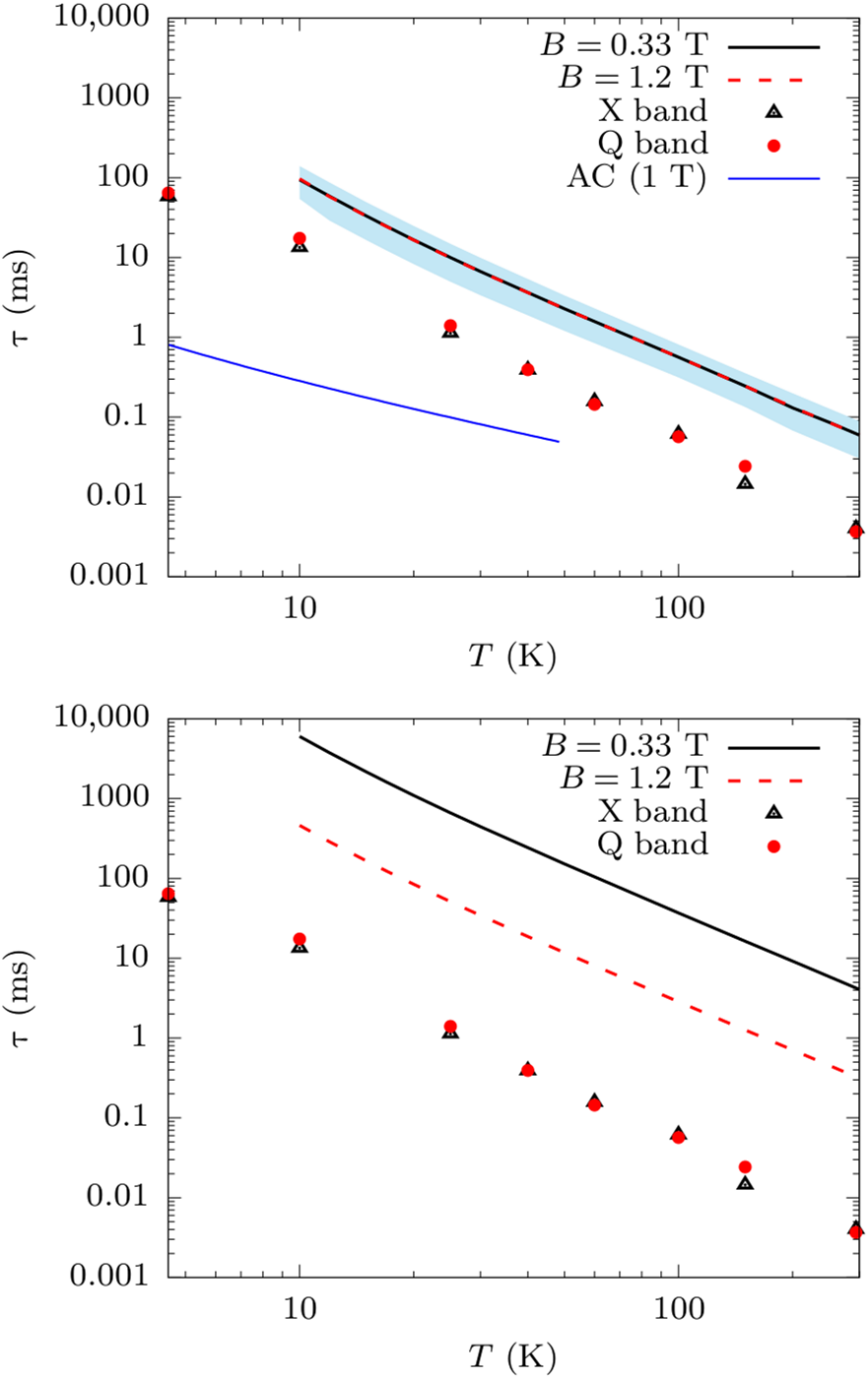
Spin-phonon relaxation time for (1). Simulated values of τ for (**1**) including the contribution of **A**-tensor (**top**) and g-tensor (**bottom**) modulations to the Raman mechanism of relaxation. Simulations are carried out for two values of the magnetic field *B* = 0.33 T (black continuous line) and *B* = 1.2 T (dashed red line) to be comparable with EPR measurements of *T*_1_ at X (black triangles) and Q bands (red squares), respectively. Results in the top panel are the average between calculations carried out with the DKH and ZORA scalar relativistic corrections, and the sky blue shade represents the two limiting values. The experimental measurement of τ from AC magnetometry at the field of 1 T for nonmagnetically diluted samples is reported in the top panel with a continuous blue line.

The magnetization dynamics of **(1)** is thus computed by solving Eqs. 4 and 7. All the secular terms are included, but no degenerate states are present because of the presence of the external fields, thus decoupling entirely population and coherence terms of the density matrix. The density matrix is initialized by applying π rotation of the electronic spin to the canonical equilibrium distribution. This initial state is chosen to mimic the experimental condition of an inversion recovery electron paramagnetic resonance (EPR) experiment used to measure *T*_1_. The time dependence of the magnetization of the electronic spin is computed to reach equilibrium following a stretched-exponential decay $M_{z}(t)=(M_{z}(0)-M_{z}^{\text{eq}})$ exp$[-{\left(t/\tau \right)}^{\beta }]+M_{z}^{\text{eq}}$ with β ∼ 0.7 − 0.9 for *A*-driven relaxation (see fig. S2) and β = 1 for *g*-driven relaxation. Figure 3 reports the comparison between the values of *T*_1_ measured with X and Q band EPR on magnetically diluted samples, together with the predicted values of τ as a function of temperature by assuming the modulation of **A** (top panel) and **g** (bottom panel) as the sole relaxation mechanisms.

Results obtained considering the modulation of the hyperfine interaction show a good agreement with inversion recovery experiments, both in terms of absolute values and external field dependence. Depending on the fine details of the DFT calculations, agreement up to a factor five and not exceeding one order of magnitude is observed. Conversely, the modulation of the Zeeman interaction is shown to overestimate spin lifetime by at least one order of magnitude. The relaxation rate due to the modulation of **g** is found to depend on the magnitude of the external field as *B*^2^, which does not fit the generally observed experimental trend for Raman relaxation in this class of complexes. For the field considered, the modulation of **A** is always the faster relaxation pathway, and the results reported in the top panel of Fig. 3 are unchanged if the modulation of **A** and **g** is simultaneously considered.

The computational results for **(1)** are in qualitative agreement with a previous study on the crystal of VO(acac)_2_ (*22*). However, differently from the VO(acac)_2_ case, *T*_1_ data obtained on diluted crystals for (**1**) are available (*36*), making it possible to demonstrate that once cross-relaxation mediated by dipolar interactions is excluded, the deviation between simulations and experiments is below one order of magnitude and the τ versus *T* profile is nicely reproduced (see top panel of Fig. 3). When nondiluted crystals are instead measured, such as in AC magnetometry experiments, a very different profile of τ versus T is often observed (see top panel of Fig. 3). The residual deviation between simulations and inversion recovery measurements is consistent with the lack of phonons outside the Γ-point and the inaccuracies of electronic structure methods (*22*). To determine the most important phonons for relaxation, we perform simulations by including only vibrations in the low-energy window and starting from the first optical mode at Γ-point, here computed at ∼12 cm^−1^. We find that the lowest-energy lying optical phonons are mainly responsible for spin relaxation even at room temperature and that phonons with energy above ∼50 cm^−1^ have no contribution (see fig. S3). This can be interpreted as an effect of thermal population, which is always larger for lowest-energy phonons. As discussed previously, low-energy vibrations of **1** are an admixture of molecular rotations and delocalized intramolecular distortions (*20*). The animation of the first optical mode at the Γ-point is reported in the Supplementary Materials.

### Spin-phonon relaxation in *S* > 1/2 systems

Next, we address the spin dynamics of **(2)** as an example of relaxation in a mononuclear transition metal complex with large zero-field splitting. This molecule contains a high-spin Co^2+^ ion that can be described with an effective *S* = 3/2 Hamiltonian

$${\hat{H}}_{s}=D{\hat{S}}_{z}^{2}+E({\hat{S}}_{x}^{2}-{\hat{S}}_{y}^{2})$$ (12)

where the large axial anisotropy term, experimentally estimated at *D* ∼ −115 cm^−1^, and vanishing rhombic term *E* remove the degeneracy of the two Kramers doublets (KDs) *M*_s_ = ±3/2 and *M*_s_ = ±1/2 (*37*, *38*). This compound crystallizes in an orthorhombic lattice with four molecules in the unit cell and eight ${\text{NHEt}}_{3}^{+}$ counter ion molecules (*37*). The spin Hamiltonian coefficients, spin-phonon coupling coefficients, and the phonons for this compound were computed in a previous work using CASSCF- and DFT-based machine learning force fields (MLFFs), respectively (*27*). The use of vibrational density of states and spin-phonon coupling intensity is reported in figs. S5 and S6.

**Fig. 4.**
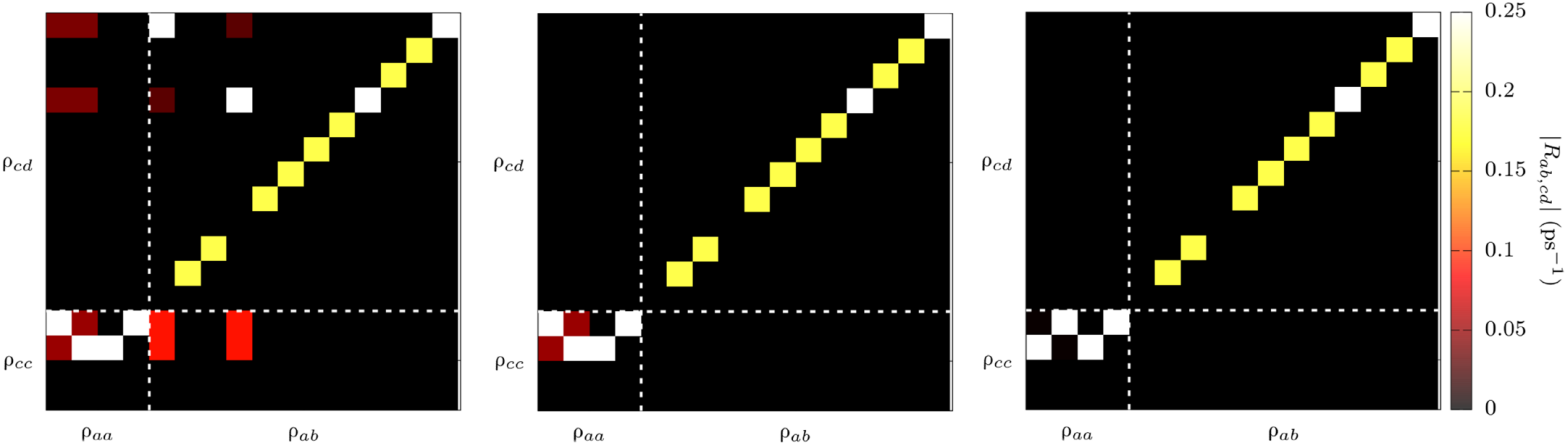
Redfield transition rates for (2). Transition rates among general elements of the density matrix predicted by the Redfield theory using the nondiagonal secular approximation and diagonal secular approximation are reported in the (**left)** and (**middle**), respectively. (**Right**) The same rates of nondiagonal secular approximation after rotating the molecular geometry to align the magnetic easy axis along *z* and applying a small external field along the same direction. The transitions rates have been computed at *T* = 40 K. The first four elements correspond to the population terms, and all the remaining elements correspond to coherence terms. All matrix elements are expressed on the basis of the spin Hamiltonian eigenstates.

We note that direct transitions within the ground-state KD (*M*_s_ = ±3/2) are prohibited by symmetry. As a consequence, both Eqs. 5 and 7 can only lead to relaxation through pathways involving excited KDs, thus involving high-energy phonons. Two-phonon contributions to Raman relaxation arising from Eq. 7 are here neglected in virtue of the fact that first-order contributions are expected to dominate. The same is not true for two-phonon contributions coming from Eq. 10, where a direct intra-Kramers double transition is made possible by the presence of virtual states. Accordingly, in our previous work, the time evolution of ${\hat{\rho }}^{s}$ was studied with the diagonal terms of Eqs. 5 and 10. However, simulations were conducted in zero field, therefore neglecting the role of coherence terms. To investigate the impact of this approximation, we compute all the terms of $R2_{\mathrm{ab},\mathrm{cd}}^{1-\text{ph}}$. The left panel of Fig. 4 shows the value of $R2_{\mathrm{ab},\mathrm{cd}}^{1-\text{ph}}$ when all terms that fulfil the condition (ω*_ac_* + ω*_db_*) = 0 are retained. We will refer to this level of approximation as nondiagonal secular approximation from now on. Instead, the central panel of Fig. 4 shows the value of $R2_{\mathrm{ab},\mathrm{cd}}^{1-\text{ph}}$ when only the terms $R2_{\mathrm{aa},\mathrm{bb}}^{1-\text{ph}}$ and $R2_{\mathrm{ab},\mathrm{ab}}^{1-\text{ph}}$ are retained. We will refer to this approximation as diagonal secular approximation from now on. As it is evident from the comparison of the left and central panels of Fig. 4, the nondiagonal secular approximation includes non-negligible terms that couple coherence and population elements of $\rho_{\mathrm{ab}}^{s}$. Last, the right panel of Fig. 4 shows that the diagonal secular approximation becomes fulfilled when Kramers degeneracy is removed by applying a small external field of 0.01 T (or smaller) along the molecule’s easy axis. We note that the eigenvalues of the matrices reported in the left and right panels of Fig. 4 are virtually identical and, at the same time, different from those of the matrix reported in the middle panel of Fig. 4. Moreover, we find that the spectrum of ${\hat{\hat{R}}2}^{1-\text{ph}}$ in the diagonal secular approximation is not rotationally invariant even in zero field, a clear indication of inconsistency.

**Fig. 5.**
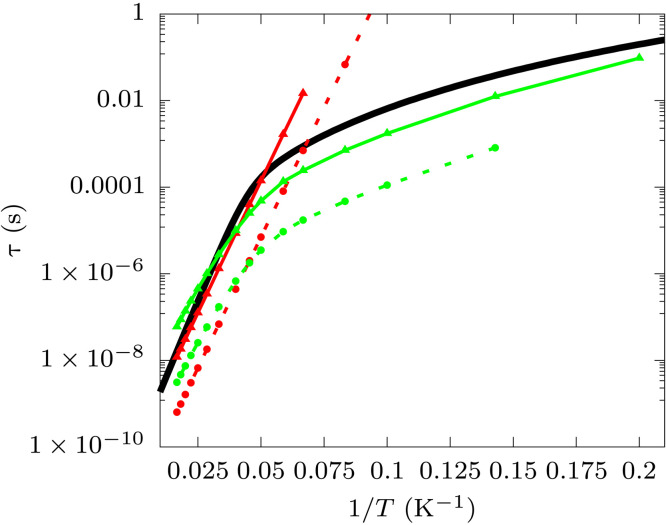
Spin-phonon relaxation time for (2). Experimental relaxation times measured at 1000 Oe with AC magnetometry are reported as black continuous lines (*38*). Simulated Orbach rates computed with the nondiagonal (continuous line and triangles) and diagonal secular approximation (dashed line and circles) are reported in red. Simulated Raman rates computed with the diagonal secular approximation with external field *B_z_* = 0.01 T (continuous line and triangles) and *B_z_* = 0.0 T (dashed line and circles) are reported in green. Raman rates in nonzero external field are computed by rotating the molecular geometry in the frame of the g-tensor’s eigenvectors.

To determine the importance of including coherence-transfer terms into the description of spin-phonon relaxation, we compute the value of spin lifetime τ for **(2)** by taking the inverse of the first nonzero eigenvalue of the matrix $R2_{\mathrm{ab},\mathrm{cd}}^{1-\text{ph}}$, as commonly done for Markov processes. The latter is equivalent to studying the decay of *M_z_*(*t*) for a molecule with the magnetization easy axis parallel to *z*. Figure 5 shows the results for the two different levels of secular approximation, together with experimental results obtained in the presence of a small external field. The application of a small external field in experiments helps in removing the effect of dipolar relaxation (*38*) not included in the simulations and therefore makes the comparison between theory and experiments more reliable. The relaxation time predicted with the diagonal secular approximation is close to the one reported before (*27*), and despite the qualitative agreement with experiments, it shows between one and two orders of magnitude of underestimation of spin lifetime. Once the nondiagonal secular approximation is introduced for one-phonon processes with the use of Eq. 5, the agreement between experimental and simulated τ becomes virtually exact in the high-*T* relaxation regime, where the Orbach mechanism drives relaxation. For temperature below 20 K, the two-phonon Raman relaxation described in Eq. 10 takes over the Orbach one. Although a nondiagonal secular approximation for this process is not available, we attempted to remove the effect of coherence transfer by applying a small external field along the molecular easy axis of magnetization. As suggested by the study of $R2_{\mathrm{ab},\mathrm{cd}}^{1-\text{ph}}$ matrix elements, the results for the diagonal and nondiagonal secular approximations become identical under these conditions (see fig. S7). This is indeed the case for **(1),** which was studied and simulated under applied field. Applying this strategy to $R4_{\mathrm{aa},\mathrm{bb}}^{2-\text{ph}}$, results improve significantly with only a negligible residual deviation left between experiments and simulations. Last, we note that simulations for **(2)** have been found extremely robust with respect to the choice of basis set, scalar relativistic effects, and dynamical correlation (see fig. S8). Moreover, predictions obtained with Γ-point phonons computed from MLFF or DFT agree very well among them, thus validating the use of MLFF (*43*) to integrate the phonons’ Brillouin zone and converge the values of τ below 10 K, where border zone and acoustic phonons start contributing (see fig. S8). As discussed elsewhere (*27*) and in agreement with what is observed here for **(1)**, low-energy phonons up to ∼50 cm^−1^ are the main drive for Raman spin relaxation in **(2)** (see fig. S9). Also in this case, it was found that these low-energy vibrations correspond to molecular rotations combined to small and delocalized intramolecular distortions (*27*).

### Spin-phonon relaxation in *J* = 15/2 lanthanide systems

**Fig. 6.**
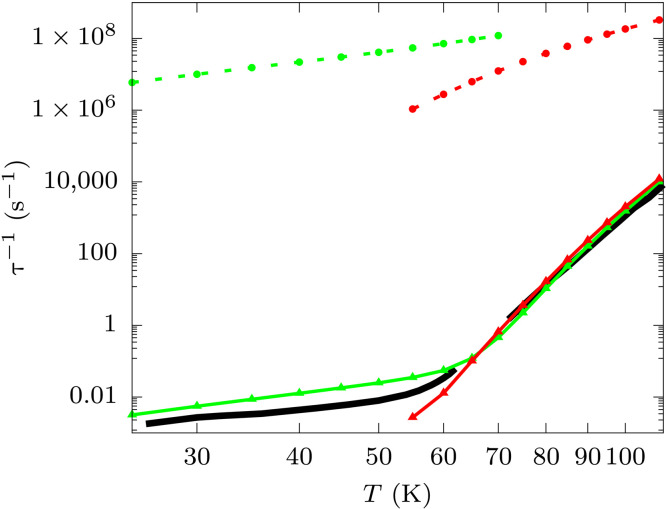
Spin-phonon relaxation time for (3). Experimental relaxation times measured in zero field with AC magnetometry and magnetization decay are reported as a black continuous line (*16*). Simulated Orbach rates computed with the nondiagonal (continuous line and triangles) and diagonal secular approximation (dashed line and circles) are reported in red. Simulated Raman rates computed with the diagonal secular approximation with external field *B_z_* = 0.01 T (continuous line and triangles) and *B_z_* = 0.0 T (dashed line and circles) are reported in green. Raman rates in the presence of nonzero external field are computed by rotating the molecular geometry in the frame of the g-tensor’s eigenvectors of the ground-state KD.

To further test the ability of our models, we extended our study to **(3)**. This molecule exhibits a Dy^3+^ ion in almost perfectly axial crystal field. This coordination stabilizes a *J* = 15/2 ground state and imposes a strong zero-field splitting that separates the ground and highest excited KDs (*Mj* = ± 15/2 and *Mj* = ± 1/2, respectively) by ∼1500 cm^−1^ (*16*). The spin states of **(3)** can be described with a generalized spin Hamiltonian (*34*, *44*)

$${\hat{H}}_{s}=\sum_{l=2,4,6}\sum_{m=-l}^{l}B_{m}^{l}{\hat{O}}_{m}^{l}(\vec{J})$$ (13)

where ${\hat{O}}_{m}^{l}(\vec{J})$ are tesseral tensor operators (*45*). **(3)** crystallizes in a triclinic unit cell with two molecular units and two [B(C_6_F_5_)_4_]^−^ (*16*) molecules as counter ions. Phonons and spin-phonon coupling coefficients were computed for **(3)** following the strategy validated on **(2)** and involves the computation of Γ-point phonons with pDFT and linear spin-phonon coupling coefficients with CASSCF. Vibrational density of states and spin-phonon coupling intensities are reported in figs. S11 to S14. Results of spin relaxation for the one-phonon Orbach process in **(3)** are reported in Fig. 6 together with experimental values. Once again, the agreement between the simulated and experimental Orbach relaxation rates is excellent, but only after the nondiagonal secular approximation is introduced. The prediction of Orbach rates obtained using the diagonal secular approximation is off by many orders of magnitude, depending on the value of *T*. Such a large disagreement is mostly due to the fact that, in the absence of the nondiagonal secular approximation, relaxation is predicted to be promoted by the first excited KD, computed at ∼477 cm^−1^, instead of being driven by absorption of phonons in resonance with the KD with energy ∼1300 cm^−1^. We tested the effect of removing Kramers degeneracy by aligning the Cartesian frame along the molecular easy axis, as expressed by the eigenvectors of ground-state KD’s *g*-tensor and by applying an external field of 0.01 T along the same direction. In agreement with what is observed for **(2)**, under these conditions, Orbach relaxation rates predicted with the diagonal and the nondiagonal secular approximations are coincident among them and with experimental relaxation rates. Exporting our computational strategy to the case of Raman relaxation, we observe a massive improvement of results, with a negligible factor of residual deviation between experiments and simulations. Differently from **(2)**, we note a slight dependence of results with respect to basis set, the number of CASSCF’s solutions considered, and the inclusion of dynamical correlation (see figs. S15 and S16), hinting to the possibility to further improve results upon a systematic exploration of these technicalities. We also note that the total relaxation time is given by the sum of Orbach and Raman relaxation rates. At temperature below 60 K, Raman relaxation is the only relevant mechanism, but at high temperature, Raman and Orbach rates are computed similar to one another, possible due to a slight overestimation of the former, and the total relaxation time is thus slightly faster than the Orbach one (see fig. S21).

Previous reports on the ab initio prediction of Orbach relaxation in **(3)** using the diagonal secular approximation are available (*16*, *28*). In those contributions, relaxation rates were overestimated by no more than two orders of magnitude, and the overall *T* profile above 70 K was correctly captured. Despite differences in the ab initio setup, we were able to qualitatively reproduce those results by using the diagonal secular approximation and the same molecular orientation of (*16*, *28*) (see fig. S17), thus validating the reproducibility of our conclusions. Turning to the analysis of phonon contributions to Raman spin relaxation in **(3)**, relaxation was found to receive contributions from several optical phonons with energy up to 80 cm^−1^ (see fig. S18). A visual inspection of these modes shows that, similarly to **(2)**, they are dominated by local molecular rotations overlapped to small intramolecular distortions. The latter mainly involve the ^t^Bu groups and the position of Dy^3+^ ion within the two Cp^−^ rings. The animation of the mode with energy ∼50 cm^−1^ is reported in the Supplementary Materials. This mode has the largest coupling among those in the sub–80 cm^−1^ energy range. Last, we determined the contribution of different excited KDs in Eq. 10 by including only one of them at the time in the simulation of τ at 40 K. This analysis shows that the first excited KD has the largest impact on Raman relaxation (see fig. S19), further pointing to the crucial role of low-energy modes as the main source of Raman relaxation.

## DISCUSSION

Although the theory of relaxation for *d*/*f* block ions has been thoroughly investigated for decades, the many parameters populating its equations have prevented its use as a predictive tool. However, once relaxation theory is combined with ab initio methods, it becomes possible to estimate spin relaxation time without any input from experiments. Such a method stands out as a key tool to benchmark our understanding of spin-phonon interaction and relaxation in an unbiased way and to provide previously unidentified strategies toward optimization of relaxation times. Nonetheless, a computational method of this complexity has many assumptions and approximations that require validation.

In this work, we have determined the main missing pieces to the puzzle of accurate spin-phonon relaxation times. The study of compound **(1)**, an isotropic *S* = 1/2 system, highlighted the importance of a careful comparison with experimental data not affected by dipolar cross-relaxation. Compounds **(2)** and **(3)** instead showed that for *S* > 1/2, Kramers system processes up to the fourth-order and accounting for coherence terms in the Redfield equations are key for obtaining quantitative predictions. We have shown how seemingly small details, such as neglecting the coherence terms of the density matrix, can lead to many orders of magnitude of deviation between predictions and experiments. As long as a parametric approach to open quantum system dynamics is used, it is impossible to detect these pitfalls, as errors are removed by rescaling factors. These results thus provide a unique proof of concept that open quantum system theory can be applied in a parameter-free fashion for complex systems such as solid state.

We envisage that further improvement of the agreement between experiments and simulations could be achieved by carefully benchmarking both electronic structure methods and the remaining theoretical approximations to the open quantum system dynamical equations. The former includes a careful study of how van der Waals (vdW) corrections to DFT affect phonons’ simulations and how much electronic structure correlation and the choice of basis set affect the prediction of spin Hamiltonian and spin-phonon coupling coefficients. From a theoretical point of view, the derivation of a full fourth-order master equation that explicitly includes the coherence terms stands out as the next fundamental challenge. Assessing the validity of the Markov approximation in situations of phonon bottleneck (*39*) and the inclusion of spin-spin interactions into the formalism are two other important directions for the field. Despite the approximations done, the methods used in this work only strongly rely on the possibility to correctly map the electronic structure of the compound of interest onto a spin Hamiltonian. This can be readily and accurately done for virtually any type of localized spin system, going from organic radicals, transition metal, or lanthanide ions to nuclear spins and solid-state defects. We argue that the molecules studied in this work, ranging from isotropic *S* = 1/2 to strongly correlated lanthanides, already cover the entire spectrum of complexity of localized magnetic systems. Moreover, we argue that the present findings also applied to situations where a spin Hamiltonian formalism breaks down. The formalism of (*18*, *19*) can be extended to include our results on quadratic and higher-order time-dependent perturbation theory, provided that an accurate-enough single-particle representation of the electronic structure theory is available.

The unprecedented level of accuracy of simulations for these different compounds also validates the theoretical framework and the ab initio methods at the same time, making it possible to confirm recent theoretical findings in terms of spin relaxation mechanism in vdW crystals of molecular Kramers system (*17*, *22*, *27*, *30*, *46*, *47*). Direct and Orbach relaxations due to resonant phonons are correctly accounted for by second-order contributions to density matrix dynamics and linear spin-phonon coupling. Two-phonon spin relaxation due to second-order contributions to density matrix dynamics and quadratic spin-phonon coupling is the main source of Raman relaxation for *S* = 1/2, while fourth-order perturbation theory and linear coupling are necessary to explain Raman relaxation in *S* > 1/2 with large uniaxial anisotropy. Simulations support the use of the expression

$$\tau^{-1}=\sum_{i}A_{i}^{1-\text{ph}}\frac{1}{\left(e^{{\beta \omega }_{i}}-1\right)}+\sum_{i}A_{i}^{2-\text{ph}}\frac{e^{{\beta \omega }_{i}}}{{\left(e^{{\beta \omega }_{i}}-1\right)}^{2}}$$ (14)

to fit one- and two-phonon contributions to experimental spin lifetime of vdW crystals of magnetic molecules (*22*). The first term in Eq. 14 can lead to either direct or Orbach relaxation depending on the energy of resonant phonons. The second term in Eq. 14 instead describes the Raman processes, which are found to be dominated by the lowest-energy phonons with a nonzero spin-phonon coupling, often in the subterahertz frequency range. This expression has been used already on a few occasions to aid the interpretation of experimental data. Restricting the sums in Eq. 14 to a few modes, whose energy and coupling are left as adjustable parameters, it was shown that additional insights on spin relaxation can be obtained with respect to a fitting done with simple power laws (*23*, *48*–*50*).

In conclusion, we have studied spin-phonon relaxation in three molecular compounds representing the most relevant classes of slow-relaxing molecular Kramers systems and have shown that open quantum system theory can be implemented in a fully ab initio fashion and used to accurately predict spin relaxation rates. Now that ab initio spin dynamics is rapidly maturing into a quantitative predictive tool, it will be possible to deploy it to study other systems such as polynuclear ion clusters (*51*), organic radicals (*6*), nuclear spins (*52*), paramagnetic defects in semiconductors (*53*), and magnetic impurities in solid-state hosts (*4*) or adsorbed on surfaces (*54*). We anticipate that our Message Passing Interface (MPI) parallel software implementation of the present formalism will facilitate it (*55*).

## MATERIALS AND METHODS

### Electronic structure simulations

The unit cell x-ray structures of **(1)** (*36*) and **(3)** (*16*) were used as starting points for a pDFT optimization with the software CP2K (*56*). DFT with the Perdew-Burke-Ernzerhof (PBE) functional (*57*), including Grimme’s D3 vdW corrections (*58*), was used together with a double-zeta polarized MOLOPT basis set. A plane wave cutoff of 2500 rydberg (Ry) and 1500 Ry were used for **(1)** and **(3)**, respectively. All the unit cell lattice force constants were computed with a two-point numerical differentiation with a step of 0.01 Å, thus providing access to only Γ-point phonons. Geometry optimization, phonons, and generation of a MLFF for **(2)** were presented before. The use of an MLFF for **(2)** provides access to super cell simulations and all the phonons of the Brillouin zone. Integration grids up to 8 × 8 × 8 were used to converge the relaxation time with respect to the *q*-point number (see fig. S10). The ORCA software (*59*) has been used for the calculation of all spin Hamiltonian terms. Simulations with ORCA were carried out on molecular geometries optimized with pDFT. The tensors **g** and **A** for **(1)** were computed with DFT using the PBE0 functional (*60*). The spin Hamiltonian of **(3)** was computed with CASSCF using a (9,7) active space and by using all the solutions with multiplicity 6, 128 solutions with multiplicity 4, and 130 solutions with multiplicity 2. Spin-orbit contributions were included through quasi-degenerate perturbation theory. ZORA and DKH scalar relativistic corrections were used for **(1)** and **(3)**, respectively. The calculation of spin-phonon coupling coefficients for **(1)** was also repeated with DKH scalar relativistic corrections. The RIJCOSX approximation with GridX6 integration grid was used for both **(1)** and **(3)**. The basis set DKH/ZORA-def2-TZVPP was used for V, S, C, and H. SARC-DKH-TZVPP was used for Dy instead. Spin-phonon coupling coefficients for **(3)** were computed using a slightly cheaper setup: State-average CASSCF simulations only included the roots with multiplicity 6, and RIJCOSX was used with the GridX4 option. The basis sets DKH-SARC-def2-QZVP, DKH-def2-TZVPP, and DKH-def2-SVP were used for Dy, C, and H, respectively. The crystal field Hamiltonian coefficients for **(3)** were obtained by fitting ORCA’s spin-orbit coupling matrix computed on the basis of CASSCF solutions. For this purpose, we used the tool getCF available as part of the MolForge software suite (*55*). Spin Hamiltonian and spin-phonon coupling coefficients for **(2)** were computed previously with CASSCF (*27*).

### Spin-phonon relaxation simulations

Phonons’ frequency and normal modes of vibrations were computed by diagonalization of the dynamical matrix

$$D_{\mathrm{ij}}(q)=\sum_{l}\Phi_{\mathrm{ij}}^{0,l}e^{iq\cdot R_{l}}$$ (15)

where **q** is a Brillouin zone vector and $\Phi_{\mathrm{ij}}^{0,l}$ is the mass-weighed lattice force constant between the degree of freedom *i* in the reference unit cell *l* = 0 and the degree of freedom *j* in the unit cell *l*. Force constants and phonons were computed starting from DFT forces with the use of the tools getFC and PhonDy available as part of the MolForge software suite (*55*). First-order spin-phonon coupling coefficients are computed with the expression

$$\left(\frac{\partial {\hat{H}}_{s}}{\partial q_{\alpha q}})=\sum_{i}^{3N}\sqrt{\frac{\hbar }{N_{q}\omega_{\alpha q}m_{i}}}L_{\alpha i}^{q}(\frac{\partial {\hat{H}}_{s}}{\partial X_{i}}\right)$$ (16)

where $L_{\alpha i}^{q}$ and $\omega_{\alpha q}^{2}$ are the eigenvectors and eigenvalues of *D_ij_*(**q**), respectively, *N_q_* is the total number of q-points used to integrate the Brillouin zone, and the sum over *i* is extended to the 3*N* molecular degrees of freedom. A similar expression is used for second-order spin-phonon coupling (*22*). The derivatives of the spin Hamiltonian terms appearing in Eq. 16 are computed by numerical differentiation. In the case of $\left(\partial B_{m}^{l}/\partial X_{i}\right)$, the parameters $B_{m}^{l}$ were computed for six distortions between ±0.05 Å for each molecular degree of freedom. Their value was then interpolated with a third-order polynomial function, where the linear coefficient corresponds to $\left(\partial B_{m}^{l}/\partial X_{i}\right)$. For the second-order differentiation of **g** and **A**, we used a four-point finite-difference expression with a step of 0.01 Å. Dirac’s delta functions appearing in the spin dynamics equations are smeared out with a Gaussian function with σ = 10 cm^−1^. A value of *i*σ is also added to the denominators of Eq. 10 to prevent possible divergence of calculated rates. Results are found to be well converged with respect to σ according to a strategy previously tested (see figs. S4 and S20) (*22*, *27*). Low-temperature results for Raman relaxation in **(2)** were also converged by sampling the vibrational Brillouin zone thanks to super cell phonons calculations (see fig. S10) (*27*).

## Supplementary Materials

### This PDF file includes:

Full expression for the second-order secular master equations Derivation of the diagonal secular fourth-order master equation Figs. S1 to S21
