# Supplementary material for: Toward exact predictions of spin-phonon relaxation times: An ab initio implementation of open quantum systems theory
Source: Sci Adv. 2022 Aug 5;8(31):eabn7880. doi: 10.1126/sciadv.abn7880 (PMC9355363; doi:10.1126/sciadv.abn7880)
Supplement: Supplementary file 1 — Full expression for the second-order secular master equations Derivation of the diagonal secular fourth-order master equation Figs. S1 to S21 [file sciadv.abn7880_sm.pdf]

Science Advances
AAAS

Supplementary Materials for

Toward exact predictions of spin-phonon relaxation times: An ab initio implementation of open quantum systems theory

Alessandro Lunghi

Corresponding author: Alessandro Lunghi, lunghia@tcd.ie

Sci. Adv. **8**, eabn7880 (2022)
DOI: 10.1126/sciadv.abn7880

The PDF file includes:

Full expression for the second-order secular master equations
Derivation of the diagonal secular fourth-order master equation
Figs. S1 to S21
Legend for data S1
Legends for movies S1 and S2

Other Supplementary Material for this manuscript includes the following:

Data S1
Movies S1 and S2

FULL EXPRESSION FOR THE SECOND-ORDER SECULAR MASTER EQUATIONS

Under the Born-Markov approximation, the second-order time-local quatum master equation leads to the Redfield equations

$$\frac{d\rho^{\mathrm{s}}_{ab}(t)}{dt} = \sum_{cd} e^{i(\omega_{ac}+\omega_{db})t} R2^{n-\mathrm{ph}}_{ab,cd}\,\rho^{\mathrm{s}}_{cd}(t)\,. \tag{1}$$

When Eq. 1 is combined with a linear spin-phonon coupling Hamiltonian, the transtion super-operator $R2^{n-\mathrm{ph}}$ becomes

$$\begin{aligned} R2^{1-\mathrm{ph}}_{ab,cd} = -\frac{\pi}{2\hbar^2}\sum_{\alpha}\Big\{ &\sum_j \delta_{bd} V^{\alpha}_{aj} V^{\alpha}_{jc} G^{1-\mathrm{ph}}(\omega_{jc},\omega_\alpha) - V^{\alpha}_{ac} V^{\alpha}_{db} G^{1-\mathrm{ph}}(\omega_{bd},\omega_\alpha) \\ &- V^{\alpha}_{ac} V^{\alpha}_{db} G^{1-\mathrm{ph}}(\omega_{ac},\omega_\alpha) + \sum_j \delta_{ca} V^{\alpha}_{dj} V^{\alpha}_{jb} G^{1-\mathrm{ph}}(\omega_{jd},\omega_\alpha)\Big\}\,, \end{aligned} \tag{2}$$

where the terms V^{α}_{ab} are a short-hand notation for $\langle a|(\partial \hat{H}_{\mathrm{s}}/\partial q_\alpha)|b\rangle$, and $|a\rangle$ and $|b\rangle$ are eigenstates of $\hat{H}_{\mathrm{s}}$. Finally, $\omega_{ab} = (E_a - E_b)/\hbar$, where E_a and E_b are eigenvalues of $\hat{H}_{\mathrm{s}}$ and $G^{1-\mathrm{ph}}$ reads

$$G^{1-\mathrm{ph}}(\omega,\omega_\alpha) = \delta(\omega-\omega_\alpha)\bar{n}_\alpha + \delta(\omega+\omega_\alpha)(\bar{n}_\alpha+1)\,, \tag{3}$$

When Eq. 1 is combined with a quadratic spin-phonon coupling Hamiltonian, the transtion super-operator $R2^{n-\mathrm{ph}}$ becomes

$$\begin{aligned} R2^{2-\mathrm{ph}}_{ab,cd} = -\frac{\pi}{4\hbar^2}\sum_{\alpha\geq\beta}\Big\{ &\sum_j \delta_{bd} V^{\alpha\beta}_{aj} V^{\alpha\beta}_{jc} G^{2-\mathrm{ph}}(\omega_{jc},\omega_\alpha,\omega_\beta) - V^{\alpha\beta}_{ac} V^{\alpha\beta}_{db} G^{2-\mathrm{ph}}(\omega_{bd},\omega_\alpha,\omega_\beta) \\ &- V^{\alpha\beta}_{ac} V^{\alpha\beta}_{db} G^{2-\mathrm{ph}}(\omega_{ac},\omega_\alpha,\omega_\beta) + \sum_j \delta_{ca} V^{\alpha\beta}_{dj} V^{\alpha\beta}_{jb} G^{2-\mathrm{ph}}(\omega_{jd},\omega_\alpha,\omega_\beta)\Big\}\,, \end{aligned} \tag{4}$$

where $V^{\alpha\beta}_{ab}$ now stands for $\langle a|(\partial^2 \hat{H}_{\mathrm{s}}/\partial q_\alpha \partial q_\beta)|b\rangle$. It should be noted that the term $\alpha = \beta$ is only included for double absorption and double emission terms. The function $G^{2-\mathrm{ph}}$ accounts for three possible processes involving two phonons: absorption of two phonons, emission of two phonons and simultaneous emission of one phonon and absorption of another one

$$G^{2-\mathrm{ph}}(\omega_{ba},\omega_\alpha,\omega_\beta) = \delta(\omega_{ba}-\omega_\beta-\omega_\alpha)\bar{n}_\beta\bar{n}_\alpha + \delta(\omega_{ba}+\omega_\beta+\omega_\alpha)(\bar{n}_\beta+1)(\bar{n}_\alpha+1)+ \tag{5}$$

$$\delta(\omega_{ba}-\omega_\beta+\omega_\alpha)\bar{n}_\beta(\bar{n}_\alpha+1) + \delta(\omega_{ba}+\omega_\beta-\omega_\alpha)(\bar{n}_\beta+1)\bar{n}_\alpha\,. \tag{6}$$

DERIVATION OF THE DIAGONAL SECULAR FOURTH-ORDER MASTER EQUATION

Given the total Hamiltonian

$$\hat{H} = \hat{H}_0 + \hat{H}_{sph} = \hat{H}_s + \hat{H}_{ph} + \hat{H}_{sph} \, , \tag{7}$$

we will refer to the eigenstates of $\hat{H}_s$ as $|a\rangle$ and to the eigenstates of $\hat{H}_{ph}$ as $|v\rangle$. Each state $|v\rangle$ will correspond to any of the possible occupations $|n_\alpha, n_\beta, ...\rangle$ of each vibrational degrees of freedom. Finally, $\hat{H}_{sph}$ will act as perturbation of these two subsets.
Perturbation theory can be used to describe the probability of observing a transitions among two different states $|i\rangle = |av\rangle$ under the effect of $\hat{H}_{sph}$. The probability to observe a transition from the state i to the state f reads

$$P_{fi} = \frac{2\pi}{\hbar} \left| \langle f|\hat{T}|i\rangle \right|^2 \delta(E_i - E_f) \, , \tag{8}$$

where E_i is the energy associated to the i-eigenstate of H_0 and the transition operator $\hat{T}$ applied to the state $|i\rangle$ reads

$$\hat{T} = \sum_{\mu=1}^{\infty} \hat{H}_{sph} \left(\frac{1}{E_i - H_0 + i0^+} \hat{H}_{sph} \right)^{\mu-1} \, . \tag{9}$$

Setting $\mu = 1$ in Eq. 9 we recover the diagonal secular contribution to the second-order matrix **R2** discussed in the main text. Setting $\mu = 2$ gives the corresponding contribution to **R4**.

Let us now explicitly write P_{fi} for $\mu = 2$. Once a complete basis set is introduced in-between the operators appearing in the definition of $\hat{T}$, the transition operator reads

$$\hat{T} = \sum_{lk} \hat{H}_{sph} |l\rangle\langle l| \frac{1}{E_i - H_0 + i0^+} |k\rangle\langle k| = \sum_{l} \hat{H}_{sph} \frac{|l\rangle\langle l|}{E_i - E_l + i0^+} \hat{H}_{sph} \, . \tag{10}$$

Introducing Eq. 10 into Eq. 8, we obtain

$$P_{fi} = \frac{2\pi}{\hbar} \left| \sum_{l} \frac{\langle f|\hat{H}_{sph}|l\rangle\langle l|\hat{H}_{sph}|i\rangle}{E_i - E_l + i0^+} \right|^2 \delta(E_i - E_f) \, . \tag{11}$$

Restricting ourselves to the linear term of $\hat{H}_{sph}$, only states that differ by no more that two phonons occupation numbers will have a non-zero transition probability and will be connected by the Hamiltonian

$$\hat{H}_{sph} = \hat{V}^\alpha \hat{q}_\alpha + \hat{V}^\beta \hat{q}_\beta \, . \tag{12}$$

Therefore, if $|i\rangle = |an_\alpha n_\beta\rangle$, the final state $|f\rangle$ can only be one of the combinations $|f\rangle = |b(n_\alpha \pm 1)(n_\beta \pm 1)\rangle$, with exclusion of $|f\rangle = |i\rangle$ that must be treated separately.

Let us begin from the case $|f\rangle = |b(n_\alpha + 1)(n_\beta + 1)\rangle$. The only non-vanishing contributions to this transition are given by the terms

$$\hat{H}_{sph} = \frac{1}{\sqrt{2}} \hat{V}^\alpha \hat{a}^\dagger_\alpha + \frac{1}{\sqrt{2}} \hat{V}^\beta \hat{a}^\dagger_\beta \, . \tag{13}$$

Introducing the last expression in 11 we obtain

$$P_{fi} = \frac{\pi}{2\hbar} \left| \sum_{l} \frac{\langle b(n_\alpha+1)(n_\beta+1)|(\hat{V}^\alpha \hat{a}^\dagger_\alpha + \hat{V}^\beta \hat{a}^\dagger_\beta)|l\rangle\langle l|(\hat{V}^\alpha \hat{a}^\dagger_\alpha + \hat{V}^\beta \hat{a}^\dagger_\beta)|an_\alpha n_\beta\rangle}{E_i - E_l + i0^+} \right|^2 \delta(E_i - E_f) \, . \tag{14}$$

For the case $\alpha \neq \beta$, there are only two possible states that contributes to the sum over l, namely $|l\rangle = |c(n_\alpha + 1)n_\beta\rangle$ and $|l\rangle = |cn_\alpha(n_\beta + 1)\rangle$, which leads to

$$P_{fi} = \frac{\pi}{2\hbar} \Big| \sum_{c} \frac{\langle b(n_\alpha+1)(n_\beta+1)|\hat{V}^\alpha \hat{a}^\dagger_\alpha|cn_\alpha(n_\beta+1)\rangle\langle cn_\alpha(n_\beta+1)|\hat{V}^\beta \hat{a}^\dagger_\beta|an_\alpha n_\beta\rangle}{E_i - E_c - n_\alpha\hbar\omega_\alpha - (n_\beta+1)\hbar\omega_\beta + i0^+} + \tag{15}$$

$$\frac{\langle b(n_\alpha+1)(n_\beta+1)|\hat{V}^\beta \hat{a}^\dagger_\beta|c(n_\alpha+1)n_\beta\rangle\langle c(n_\alpha+1)n_\beta|\hat{V}^\alpha \hat{a}^\dagger_\alpha|an_\alpha n_\beta\rangle}{E_i - E_c - (n_\alpha+1)\hbar\omega_\alpha - n_\beta\hbar\omega_\beta + i0^+} \Big|^2 \delta(E_i - E_f) \, . \tag{16}$$

Finally, applying the creating operators to the vibrational part of the H_0 eigenstates

$$P_{fi} = \frac{\pi}{2\hbar}\Big|\sum_c \frac{\langle b|\hat{V}^\alpha|c\rangle\langle c|\hat{V}^\beta|a\rangle}{E_a - E_c - \hbar\omega_\beta + i0^+} + \frac{\langle b|\hat{V}^\beta|c\rangle\langle c|\hat{V}^\alpha|a\rangle}{E_a - E_c - \hbar\omega_\alpha + i0^+}\Big|^2 (n_\alpha + 1)(n_\beta + 1)\delta(E_a - E_b - \hbar\omega_\alpha - \hbar\omega_\beta) = , \quad (17)$$

or equivalently

$$P_{fi} = \frac{\pi}{2\hbar^2}\Big|\sum_c \frac{\langle b|\hat{V}^\alpha|c\rangle\langle c|\hat{V}^\beta|a\rangle}{E_c - E_a - \hbar\omega_\beta - i0^+} + \frac{\langle b|\hat{V}^\beta|c\rangle\langle c|\hat{V}^\alpha|a\rangle}{E_c - E_a - \hbar\omega_\alpha - i0^+}\Big|^2 (n_\alpha + 1)(n_\beta + 1)\delta(\omega_{ba} + \omega_\alpha + \omega_\beta) . \quad (18)$$

Finally, in order to obtain the probability to observe a transition among the sole spin states a and b, regardless of the phonon states, we need to sum over all possible initial vibrational states and multiply by their probability. This lead to

$$W^{++}_{ba}(\alpha\beta) = \frac{\pi}{2\hbar^2}\Big|\sum_c \frac{\langle b|\hat{V}^\alpha|c\rangle\langle c|\hat{V}^\beta|a\rangle}{E_c - E_a - \hbar\omega_\beta - i0^+} + \frac{\langle b|\hat{V}^\beta|c\rangle\langle c|\hat{V}^\alpha|a\rangle}{E_c - E_a - \hbar\omega_\alpha - i0^+}\Big|^2 (\bar{n}_\alpha + 1)(\bar{n}_\beta + 1)\delta(\omega_{ba} + \omega_\alpha + \omega_\beta) , \quad (19)$$

where $\bar{n}_\alpha$ is now the thermal population of a vibrational state as expressed by the Bose-Einstein statistics.

In the case $\alpha = \beta$, only the single term $|l\rangle = |c(n_\alpha + 1)\rangle$ would contribute to the summation over l in Eq. 14. Therefore, if Eq.18 is employed, it should be multiplied by a factor 1/4 for $\alpha = \beta$ to avoid double counting of contributions.

The case of transitions to the final states of the form $|f\rangle = |b(n_\alpha - 1)(n_\beta - 1)\rangle$ follows exactly the same route of $|f\rangle = |b(n_\alpha + 1)(n_\beta + 1)\rangle$, leading to the expression

$$W^{--}_{ba}(\alpha\beta) = \frac{\pi}{2\hbar^2}\Big|\sum_c \frac{\langle b|\hat{V}^\alpha|c\rangle\langle c|\hat{V}^\beta|a\rangle}{E_c - E_a + \hbar\omega_\beta - i0^+} + \frac{\langle b|\hat{V}^\beta|c\rangle\langle c|\hat{V}^\alpha|a\rangle}{E_c - E_a + \hbar\omega_\alpha - i0^+}\Big|^2 \bar{n}_\alpha\bar{n}_\beta\delta(\omega_{ba} - \omega_\alpha - \omega_\beta) . \quad (20)$$

Let us now investigate the case $|f\rangle = |b(n_\alpha + 1)(n_\beta - 1)\rangle$. In this case, The only non-vanishing contributions to the transition involving the states $|l\rangle$ are given by the terms

$$\hat{H}_{sph} = \frac{1}{\sqrt{2}}\hat{V}^\alpha(\hat{a}^\dagger_\alpha + \hat{a}_\alpha) + \frac{1}{\sqrt{2}}\hat{V}^\beta(\hat{a}^\dagger_\beta + \hat{a}_\beta) . \quad (21)$$

Following a similar strategy as before and with a little algebra, one can derive that the only contributing terms are

$$\langle f|a^\dagger_\alpha|l\rangle\langle l|a_\beta|i\rangle + \langle f|a_\beta|l\rangle\langle l|a^\dagger_\alpha|i\rangle . \quad (22)$$

Differently from the double absorption/emission case, here two terms also appear for the case $\alpha = \beta$.
Form Eq. 22 follows that

$$W^{+-}_{ba}(\alpha\beta) = \frac{\pi}{2\hbar^2}\Big|\sum_c \frac{\langle b|\hat{V}^\alpha|c\rangle\langle c|\hat{V}^\beta|a\rangle}{E_c - E_a - \hbar\omega_\beta - i0^+} + \frac{\langle b|\hat{V}^\beta|c\rangle\langle c|\hat{V}^\alpha|a\rangle}{E_c - E_a + \hbar\omega_\alpha - i0^+}\Big|^2 (\bar{n}_\alpha + 1)\bar{n}_\beta\delta(\omega_{ba} + \omega_\alpha - \omega_\beta) . \quad (23)$$

Now that we have derived an expression for all the possible two-phonon transitions due to fourth-order time dependent perturbation theory we can combined them and sum over all possible pairs of vibrational degrees of freedom in order to obtain the final probability of observing a spin transition $|a\rangle \to |b\rangle$

$$W_{ba} = R4^{2-\mathrm{ph}}_{bb,aa} = \frac{\pi}{2\hbar^2} \sum_{\alpha \geq \beta} \left[A_{\alpha\beta} W^{--}_{ba}(\alpha\beta) + A_{\alpha\beta} W^{++}_{ba}(\alpha\beta) + B_{\alpha\beta} W^{+-}_{ba}(\alpha\beta) + B_{\alpha\beta} W^{-+}_{ba}(\alpha\beta) \right] \text{ , and} \tag{24}$$

$$W^{--}_{ba}(\alpha\beta) = \Big| \sum_c \frac{\langle b|\hat{V}_\alpha|c\rangle\langle c|\hat{V}_\beta|a\rangle}{E_c - E_a - \hbar\omega_\beta} + \frac{\langle b|\hat{V}_\beta|c\rangle\langle c|\hat{V}_\alpha|a\rangle}{E_c - E_a - \hbar\omega_\alpha} \Big|^2 \bar{n}_\alpha \bar{n}_\beta \delta(\omega_{ba} - \omega_\alpha - \omega_\beta) \tag{25}$$

$$W^{++}_{ab}(\alpha\beta) = \Big| \sum_c \frac{\langle b|\hat{V}_\alpha|c\rangle\langle c|\hat{V}_\beta|a\rangle}{E_c - E_a + \hbar\omega_\beta} + \frac{\langle b|\hat{V}_\beta|c\rangle\langle c|\hat{V}_\alpha|a\rangle}{E_c - E_a + \hbar\omega_\alpha} \Big|^2 (\bar{n}_\alpha + 1)(\bar{n}_\beta + 1) \delta(\omega_{ba} + \omega_\alpha + \omega_\beta) \tag{26}$$

$$W^{+-}_{ab}(\alpha\beta) = \Big| \sum_c \frac{\langle b|\hat{V}_\alpha|c\rangle\langle c|\hat{V}_\beta|a\rangle}{E_c - E_a + \hbar\omega_\beta} + \frac{\langle b|\hat{V}_\beta|c\rangle\langle c|\hat{V}_\alpha|a\rangle}{E_c - E_a - \hbar\omega_\alpha} \Big|^2 \bar{n}_\alpha (\bar{n}_\beta + 1) \delta(\omega_{ba} - \omega_\alpha + \omega_\beta) \tag{27}$$

$$W^{-+}_{ab}(\alpha\beta) = \Big| \sum_c \frac{\langle b|\hat{V}_\alpha|c\rangle\langle c|\hat{V}_\beta|a\rangle}{E_c - E_a - \hbar\omega_\beta} + \frac{\langle b|\hat{V}_\beta|c\rangle\langle c|\hat{V}_\alpha|a\rangle}{E_c - E_b + \hbar\omega_\alpha} \Big|^2 (\bar{n}_\alpha + 1) \bar{n}_\beta \delta(\omega_{ba} + \omega_\alpha - \omega_\beta) \, , \tag{28}$$

where the coefficient $A_{\alpha\beta} = (1 - 3/4\delta_{\alpha\beta})$ takes care of cancelling double counting for $\alpha = \beta$ in the double absorption/emission transitions, and the coefficient $B_{\alpha\beta} = (1 - 1/2\delta_{\alpha\beta})$ eliminates the double counting of the transition $(W^{+-}_{ba}(\alpha\alpha) + W^{-+}_{ba}(\alpha\alpha))$.

It is important to remark that Eq. 28 should also include additional terms that cancel the divergence of the denominators [see Ref 8 and 9 of the main manuscript]. We neglect those terms in light of the fact that the numerical sparsity of the phonon spectrum never leads to such divergences.

SUPPLEMENTARY DATA

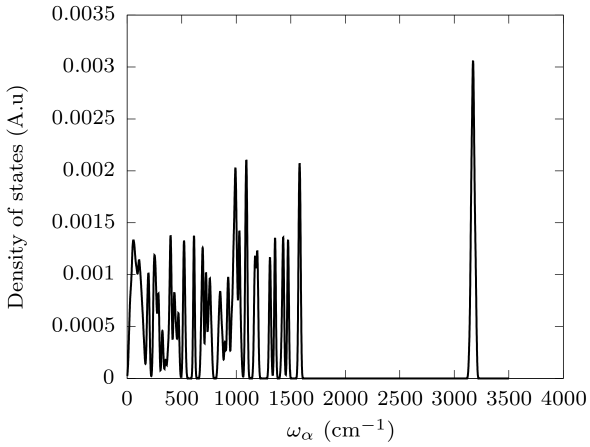

Fig. S 1. **Phonons density of states.** Vibrational density of states of **(1)** computed with Gaussian smearing of 10 cm^{-1}.

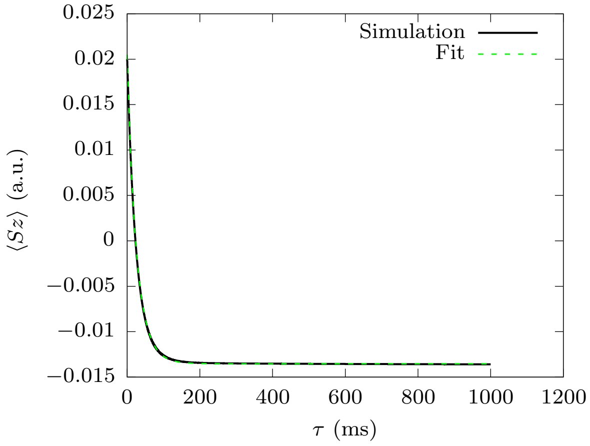

Fig. S 2. **Magnetization dynamics.** Simulated magnetization for **(1)** as function of time at 40 K (black continuos line) overlapped to a fit with an exponential function (dashed green line).

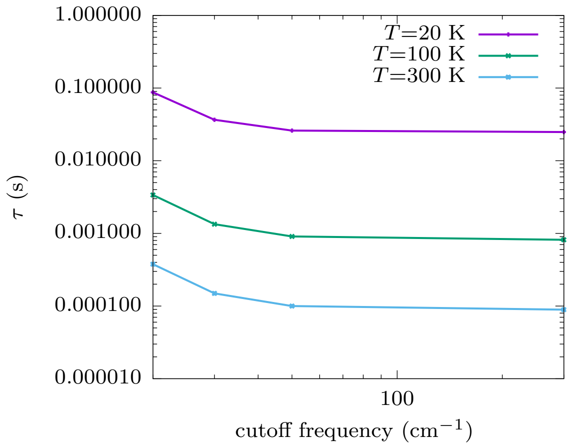

Fig. S 3. **Relaxation time as function of phonon energy cutoff.** The relaxation time of τ for **(1)** is computed at 20, 100, and 300 K including phonons up to a cutoff value of energy.

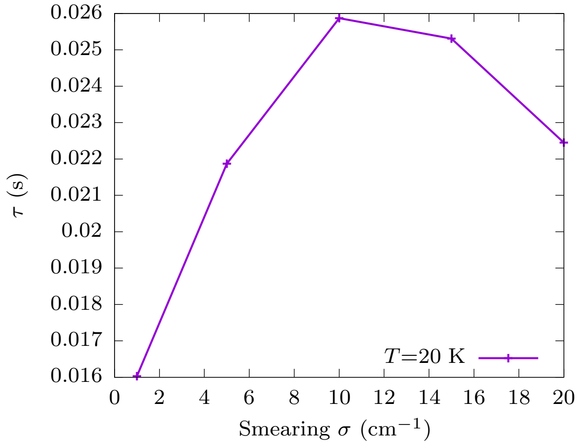

Fig. S 4. **Relaxation time as function of Gaussian smearing.** The relaxation time of τ for **(1)** is computed at 20 K for different values of Gaussian smearing σ.

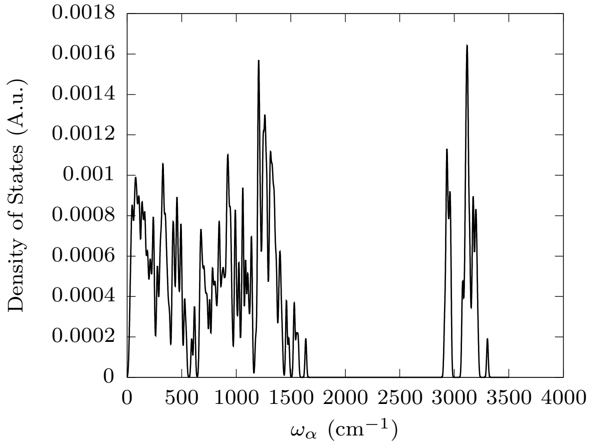

Fig. S 5. **Phonons density of states.** Vibrational density of states of **(2)** computed with Gaussian smearing of 10 cm^{-1}.

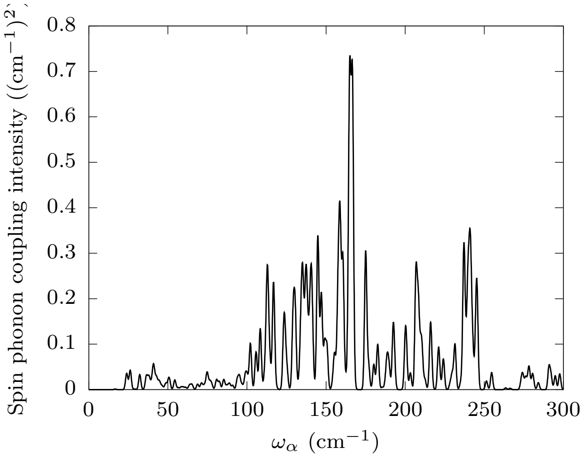

Fig. S 6. **Spin-phonons coupling intensity.** Spin-phonon coupling intensity is computed for **(2)** as $\sum_{ij} |\partial D_{ij}/\partial q_\alpha|^2$ and reported as function of phonons' frequency. A Gaussian smearing of 1 cm^{-1} is applied.

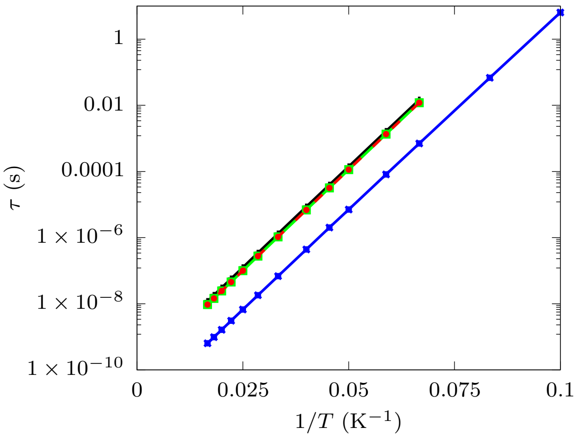

Fig. S 7. **Dependence of relaxation time on the secular approximation.** Values of τ for **(2)** computed with the non-diagonal secular approximation for an arbitrary molecular orientation and $B = 0$ T (black continuos line), and by orienting the molecule's easy axis along z and by applying $B_z = 0.01$ T (gree continuos line). Results for the secular approximation and with the molecule oriented arbitrarily and $B = 0$ T are reported with a blue continuos line. Results for the secular approximation with the molecule oriented such as the easy axis is parallel to z and with $B = 0.01$ T are reported with a dashed red line.

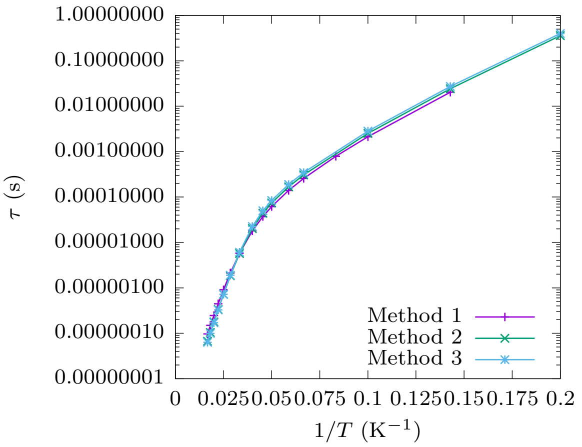

Fig. S 8. **Dependence of relaxation time on the static spin Hamiltonian.** Values of Raman relaxation τ for **(2)** are computed employing different static spin Hamiltonias, each computed with a different electronic structure methods. Method 1 is the same as reported in the main manuscript. Method 2 uses NEVPT2 on top of CASSCF. Method 3 also includes DKH scalar relativistic corrections.

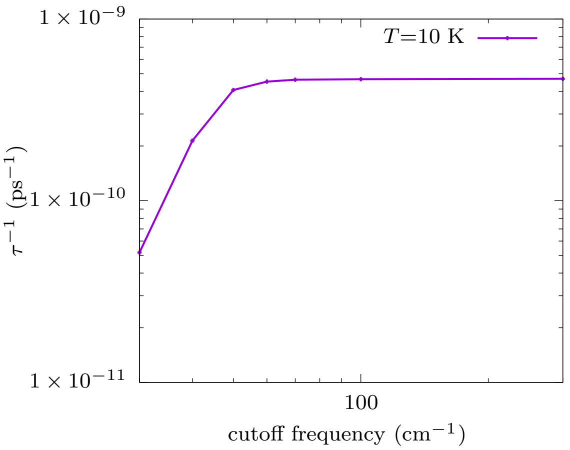

Fig. S 9. **Relaxation time as function of phonon energy cutoff.** The Raman relaxation time of τ for **(2)** is computed at 10 K including phonons up to a cutoff value of energy.

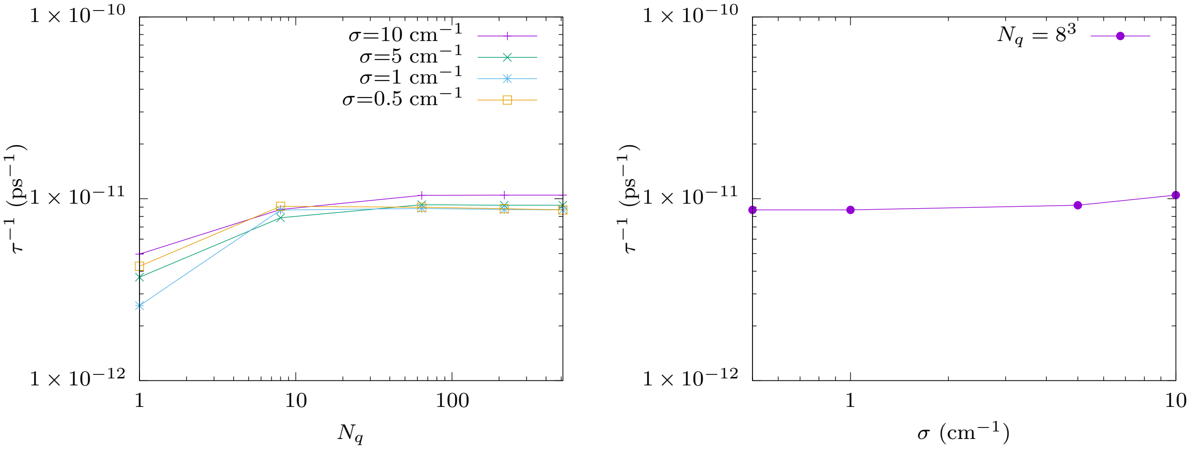

Fig. S 10. **Convergence of relaxation time and smearing.** The Raman relaxation time of τ for **(2)** is computed at 5 K for different values of Gaussian smearing (σ)and number of q-points (N_q) for integrating the Brillouin zone.

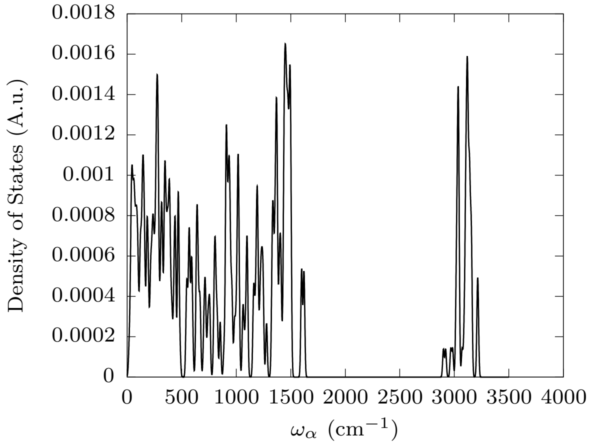

Fig. S 11. **Phonons density of states.** Vibrational density of states of **(2)** computed with Gaussian smearing of 10 cm^{-1}.

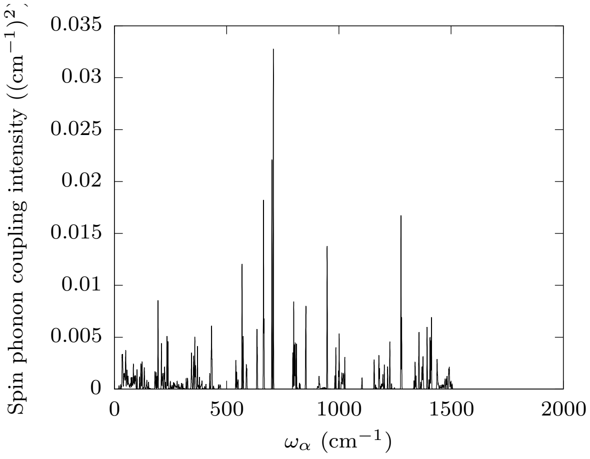

Fig. S 12. **Spin-phonons coupling intensity for B^2_m.** Spin-phonon coupling intensity is computed for **(3)** as $\sum_m |\partial B^2_m/\partial q_\alpha|^2$ and reported as function of phonons' frequency. A Gaussian smearing of 1 cm^{-1} is applied.

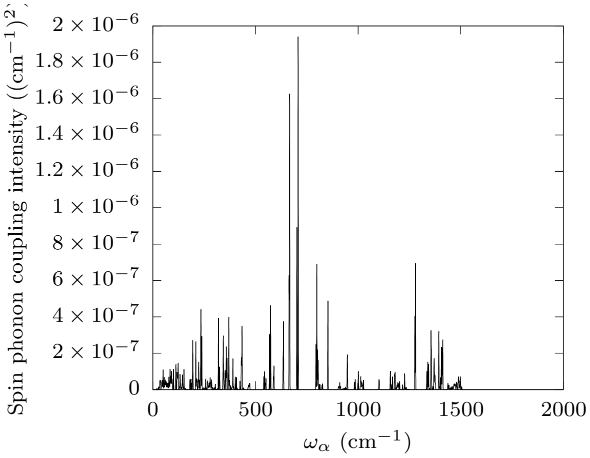

Fig. S 13. **Spin-phonons coupling intensity for B^4_m.** Spin-phonon coupling intensity is computed for **(3)** as $\sum_m |\partial B^4_m/\partial q_\alpha|^2$ and reported as function of phonons' frequency. A Gaussian smearing of 1 cm^{-1} is applied.

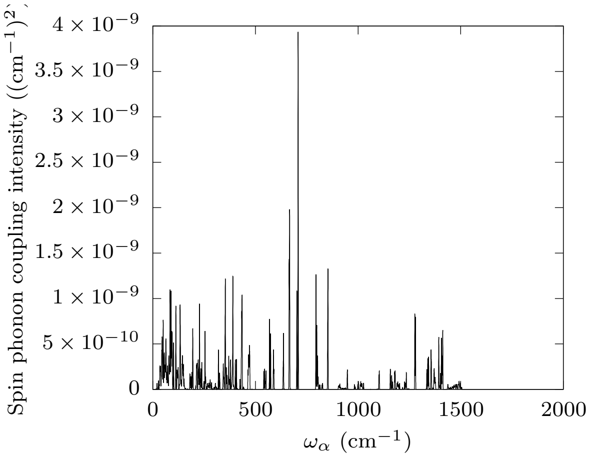

Fig. S 14. **Spin-phonons coupling intensity for B^6_m.** Spin-phonon coupling intensity is computed for **(3)** as $\sum_m |\partial B^6_m/\partial q_\alpha|^2$ and reported as function of phonons' frequency. A Gaussian smearing of 1 cm^{-1} is applied.

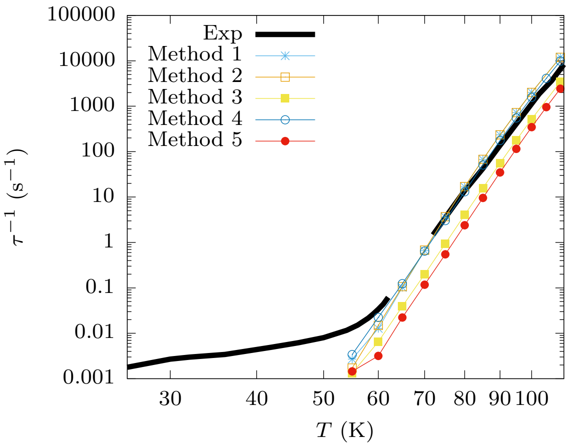

Fig. S 15. **Dependence of Orbach relaxation time on the static spin Hamiltonian.** Values of τ for **(3)** are reported for a series of calculations that uses different static spin Hamiltonian operators. The latter are obtained from different variants of the electronic structure setup. Method 1 corresponds to the one used in the main manuscript. Method 2 replaced the use of RIJCOSX with RIJK. Method 3 uses only CASSCF roots with multiplicity six. Method 4 uses only CASSCF roots with multiplicity six, includes NEVPT2, and the def2-QZVP basis set for Dy. Method 5 uses only CASSCF roots with multiplicity six and includes NEVPT2. The continuous black line reports experimental values.

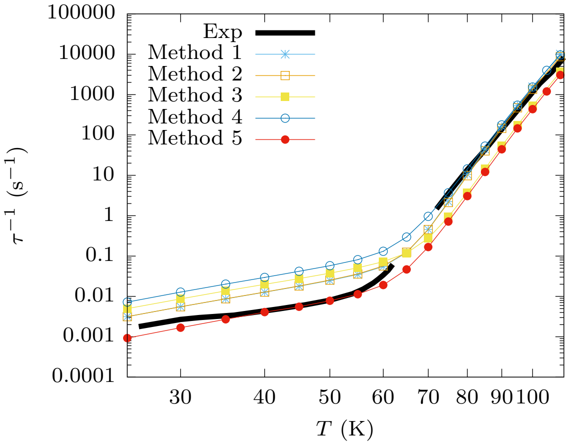

Fig. S 16. **Dependence of Raman relaxation time on the static spin Hamiltonian.** Values of τ for **(3)** are reported for a series of calculations that uses different static spin Hamiltonian operators. The latter are obtained from different variants of the electronic structure setup. Method 1 corresponds to the one used in the main manuscript. Method 2 replaced the use of RIJCOSX with RIJK. Method 3 uses only CASSCF roots with multiplicity six. Method 4 uses only CASSCF roots with multiplicity six, includes NEVPT2, and the def2-QZVP basis set for Dy. Method 5 uses only CASSCF roots with multiplicity six and includes NEVPT2. The continuous black line reports experimental values.

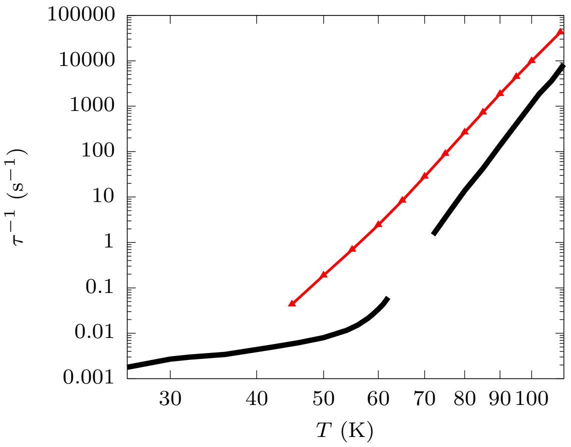

Fig. S 17. **Comparison with previous results for DyCp.** Simulations for Orbach relaxation were carried out using the diagonal secular approximation and the same molecular orienttion as in Nature, 548, 439–442 (2017) and JACS, 143, 5943–5950 (2021).

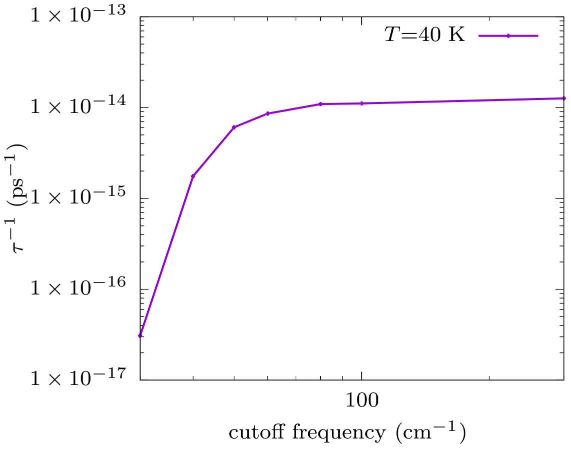

Fig. S 18. **Relaxation time as function of phonon energy cutoff.** The relaxation time of τ for **(3)** is computed at 40 K including phonons up to a cutoff value of energy.

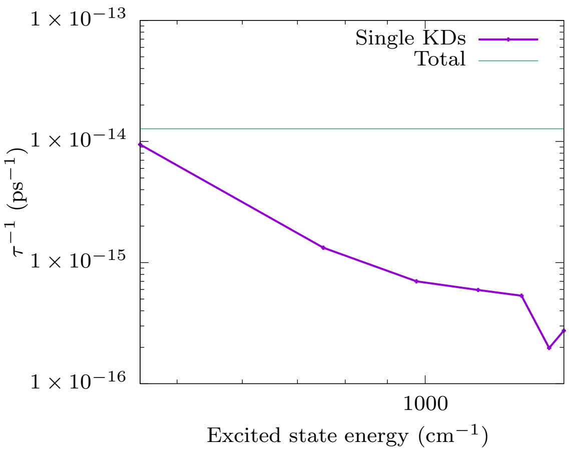

Fig. S 19. **Relaxation time as function of excited KDs.** The relaxation time of τ for **(3)** is computed at 40 K including different KDs. Results are ported as function of the energy of the KD included in the simulation. The green line reports the value obtained by including all the KDs.

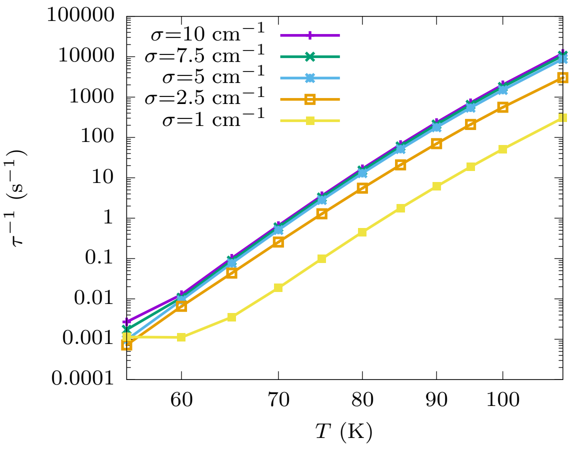

Fig. S 20. **Relaxation time as function of Gaussian smearing.** The Orbach relaxation time of τ for **(3)** is computed for different values of Gaussian smearing σ.

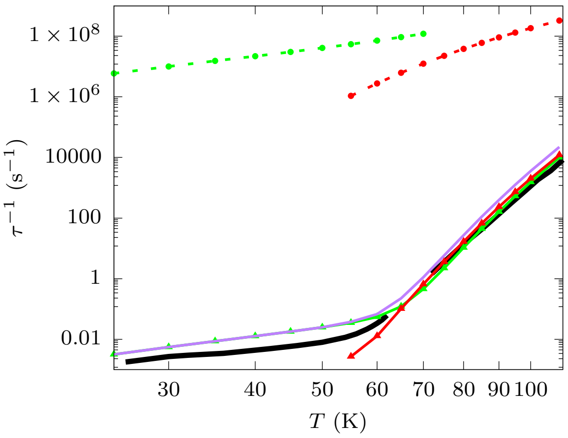

Fig. S 21. **Total relaxation time.** Experimental relaxation times measured in zero field with AC magnetometry and magnetization decay are reported as a black continuous line as taken from Ref. 16 of the main manuscript. Simulated Orbach rates computed with the non-diagonal (continuous line and triangles) and diagonal secular approximation (dashed line and circles) are reported in red. Simulated Raman rates computed with the diagonal secular approximation with external field $B_z = 0.01$ Tesla (continuous line and triangles) and $B_z = 0.0$ Tesla (dashed line and circles) are reported in green. Raman rates in the presence of non-zero external field are computed by rotating the molecular geometry in the frame of the g-tensor's eigenvectors of the ground-state KD. The purple line represents the total relaxation rate computed wiht the non-diagonal secular approximation, *i.e.* the sum or Orbach and Raman contributions.

Data S 1. **Molforge software source code.** Molforge v1.0.1_beta was used to perform all the spin dynamics simulations reported in the manuscript and its source code is provided. Molforge used The code is maintained and updated at https://github.com/LunghiGroup/MolForge.

Movie S 1. **Optical vibration in (1)** The first optical mode at the Γ-point for (1) is reported as animation.

Movie S 2. **Optical vibration in (3)** The Γ-point optical mode with energy of 50 cm^{-1} for (3) is reported as animation.
