# Supplementary figures and images for: Toward exact predictions of spin-phonon relaxation times: An ab initio implementation of open quantum systems theory
Source: Sci Adv. 2022 Aug 5;8(31):eabn7880. doi: 10.1126/sciadv.abn7880 (PMC9355363; doi:10.1126/sciadv.abn7880)

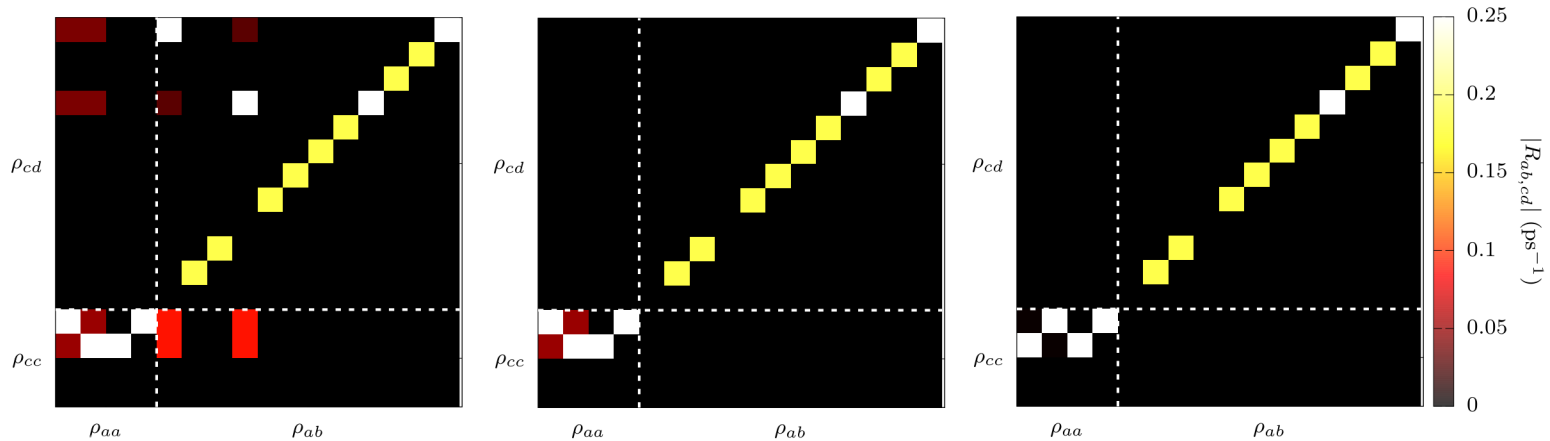

Supplement: Supplementary file 2 — Data S1 [file sciadv.abn7880_data_s1.zip › tau_CoL2_maptot2.pdf]

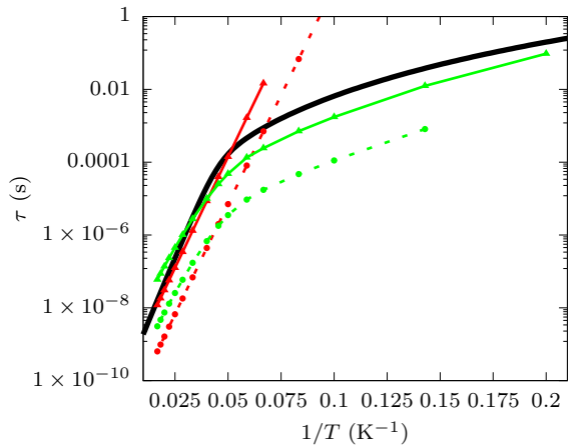

Supplement: Supplementary file 2 — Data S1 [file sciadv.abn7880_data_s1.zip › tau_CoL2_ML_tot.pdf]

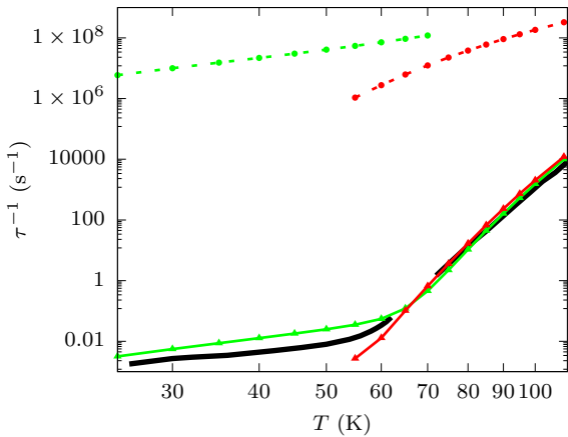

Supplement: Supplementary file 2 — Data S1 [file sciadv.abn7880_data_s1.zip › tau_DyCp_tot_final.pdf]

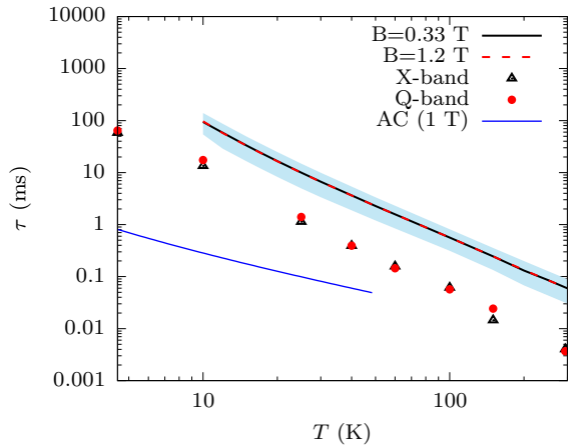

Supplement: Supplementary file 2 — Data S1 [file sciadv.abn7880_data_s1.zip › tau_VOdmit2_ddA_AC.pdf]

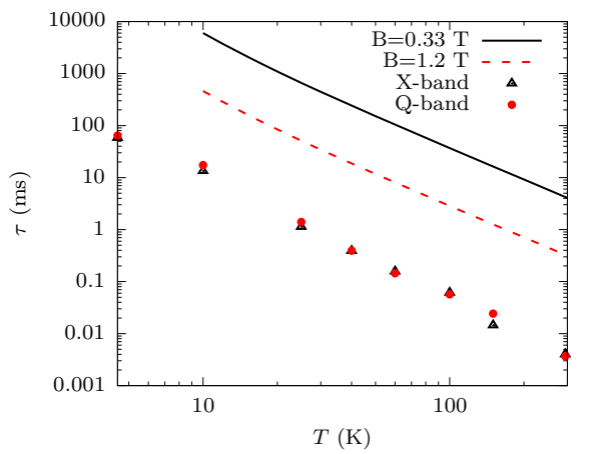

Supplement: Supplementary file 2 — Data S1 [file sciadv.abn7880_data_s1.zip › tau_VOdmit2_ddG.pdf]
